# Conceptualising and operationalising ‘patient and public involvement’ (PPI) and ‘responsible research and innovation’ (RRI) in youth mental health research: a scoping review

**DOI:** 10.1186/s40900-026-00969-3

**Published:** 2026-09-24

**Authors:** Josimar Antônio de Alcântara Mendes, Mathijs Lucassen, Alex Adams, Ayan Mahamud, Lucy Hitcham, Sachiyo Ito-Jaeger, Christine Aicardi, Emma Nielsen, Lily Roberts, Ellen Townsend, Marina Jirotka

**Affiliations:** 1 Responsible Technology Institute, Department of Computer Science, University of Oxford, Wolfson Building, Parks Rd, Oxford, OX1 3QG UK; 2 City St George’s, University of London, Northampton Square, London, EC1V 0HB UK; 3 Sprouting Minds (Young People Advisory Group – Digital Youth Programme), University of Nottingham, Nottingham, NG7 2UH UK; 4 University of Nottingham, Nottingham, NG7 2UH UK; 5 National Institute for Health and Care Research (NIHR) Nottingham Biomedical Research Centre, Nottingham, NG7 2UH UK; 6 Mental Health & Clinical Neuroscience, School of Medicine, The University of Nottingham, Nottingham, NG7 2UH UK; 7 King’s College London, Strand, London, WC2R 2LS UK; 8 EPSRC Horizon Centre for Doctoral Training, University of Nottingham, Nottingham, United Kingdom

**Keywords:** Patient and Public Involvement (PPI), Responsible Research and Innovation (RRI), Adolescent Mental Health, Youth Involvement, Co-production, Collaborative research

## Abstract

**Background:**

Meaningful youth involvement has gained attention in mental health research, especially in collaborative approaches such as Patient and Public Involvement (PPI) and Responsible Research and Innovation (RRI). While both aim to support inclusive research, there is limited clarity on how they are conceptualised and used in projects together with young people. This scoping review examines how PPI and RRI approaches are defined and implemented in mental health research involving young people as collaborators rather than solely as research participants.

**Methods:**

The included studies were empirical, peer-reviewed articles published in English that involved young people (aged 10–25 years) as collaborators in shaping mental health-related research, interventions, or products beyond their contributions as study participants, and used PPI, RRI, or related collaborative approaches. Reference to terms such as “participatory research” alone was not treated as evidence of involvement; eligibility required young people to have a reported collaborative role. Eight bibliographic databases (Scopus, Web of Science, MEDLINE, PsycINFO, CINAHL, ASSIA, PsycARTICLES, and IBSS) were searched on 21 February 2024. Following the JBI methodology and PRISMA-ScR guidelines, records were screened. Data were then charted from the included papers to capture study characteristics, definitions and applications of PPI and RRI, and the modes and reported forms or roles of youth involvement.

**Results:**

Thirty-two papers were included, most were published from 2022 onwards, originated from high-income English-speaking countries, and defined and applied PPI more frequently than RRI. However, both approaches lacked operational clarity. Key terms such as co-production, co-design, and participatory research were widely but inconsistently used, often without clear definitions or sufficient information to establish whether young people were involved as partners rather than solely as research participants. Explicit discussions related to power or intersectional barriers to youth involvement were rare. Some studies reported involving large numbers of young people, including up to 339 youths, raising questions about feasibility and depth of involvement.

**Conclusions:**

This review highlights promising trends in extending youth involvement in mental health research, but also reveals important conceptual and ethical limitations. The findings emphasise the need for clearer definitions, explicit distinctions between meaningful youth involvement and being researched as a study’s participant, more inclusive research practices, and stronger commitments to equity. Expanding future research is crucial for advancing responsible and collaborative approaches in youth mental health.

**Supplementary Information:**

The online version contains supplementary material available at https://doi.org/10.1186/s40900-026-00969-3.

## Abstract (plain language summary)

Many mental health studies are about young people, but young people are not always meaningfully involved in shaping the research. This review looked at published studies where young people aged 10 to 25 helped with mental health-related research, such as planning studies, designing services or tools, giving advice, or working as partners. We wanted to understand how researchers describe this involvement and what roles young people are given. We identified 32 relevant studies. Most came from high-income English-speaking countries, especially the United Kingdom, Australia, Canada, and the United States. Many studies used words such as “co-design”, “co-production”, or “participation”, but these words were often not clearly explained. This makes it difficult to know how much influence young people actually had. Some studies involved many young people, but did not always explain whether they had real decision-making power. The review also found that young people were not always clearly recognised for their contributions, for example by being named as authors when they had helped shape and carry out the actual work. Overall, this review shows that involving young people in mental health research is becoming more common, but researchers need to be clearer about what involvement means, how young people are supported, how power is shared, and how young people’s contributions are recognised.

**Supplementary Information:**

The online version contains supplementary material available at https://doi.org/10.1186/s40900-026-00969-3.

## Background

Adolescence and early adulthood represent critical developmental periods during which mental health conditions frequently emerge. Epidemiological studies indicate that 62.5% of mental health disorders begin before the age of 25 years, highlighting the critical need to support early identification of these conditions as well as effective interventions [1]. Involving end-users, such as young people, throughout an intervention-related research project is vital to maximising its relevance and impact [2]. That is because the lived experience of young people offers essential insights into their needs and contexts, making their involvement critical to research efforts. While interest in youth involvement in research is growing, researchers often struggle to engage young people in meaningful and sustained ways [3]. As a result, the literature on collaborative efforts involving young people in mental health, whether in academic research, intervention design, or product development, remains limited, especially in terms of the depth and quality of youth involvement [4].

Approaches such as Patient and Public Involvement (PPI) and Responsible Research and Innovation (RRI) have been introduced to help guide health-related initiatives, aiming to harness the unique perspectives of young people [5–8]. Their relevance is particularly important in the youth mental health field because conventional researcher-led approaches may position young people solely as research participants and privilege priorities, assumptions, and interpretations defined by selected adults or professionals [8–11]. Consequently, research questions, methods, and interventions may inadequately reflect young people’s lived experience, developmental needs, cultural contexts, and everyday realities, potentially reducing the relevance, acceptability, and feasibility of the research undertaken. In this scenario, meaningful involvement^1^ can help address these limitations by enabling young people to shape research priorities as well as a study’s design, implementation, interpretation, and eventual dissemination. In turn, this may enhance the contextual and cultural responsiveness of research, strengthen the acceptability of interventions intended for youths, support their implementation, and increase the likelihood that they produce outcomes that are relevant and beneficial to young people [3, 4, 8, 9, 12–20].

However, the ways in which these approaches are defined and implemented vary considerably across academic disciplines, funders and projects, ranging from a brief, one-off consultation to sustained, shared decision-making over the life cycle of a project, with the latter generally representing a deeper level of involvement [12–14, 21–23]. This breadth of interpretation in terms of what constitutes PPI and RRI makes it difficult to compare studies, evaluate quality, or distinguish between genuinely collaborative work and more tokenistic, ‘tick-box’ exercises, thereby blurring what meaningful involvement should ideally look like in practice. This involvement matters, as Article 12 of the United Nations Convention on the Rights of the Child affirms the right of children and young people to freely express their views on matters affecting them [24, 25]. PPI and RRI are therefore extremely valuable approaches in terms of upholding this right, yet they are often applied without a clear articulation of their respective aims, scope, and added value. As a consequence, they are sometimes mistakenly treated as interchangeable labels rather than distinct, complementary approaches, which limits their potential to guide robust, youth-centred practice. Clarifying and expanding how these concepts are understood is therefore essential for setting coherent expectations and for strengthening the foundations of responsible youth involvement in mental health research.

Throughout this review, PPI and RRI are treated as broad umbrella approaches rather than as single, prescriptive approaches. We will use the term “concept” when discussing how PPI or RRI is defined and understood, “approach” when referring to its overarching orientation and application, and “framework” only for structured models or tools through which an approach may be operationalised, such as in relation to AREA (Anticipate, Reflect, Engage, Act) within RRI. This terminology is adopted as an analytical convention for clarity, while recognising that these terms are themselves used inconsistently across the literature.

### Patient and public involvement (PPI)

In health research, recognition of the importance of involving patients and the public has been steadily increasing [13, 14, 23, 26], with health research funders requiring more evidence that beneficiaries or end users have contributed to the development of studies [27]. In the UK, this is commonly known as PPI which is defined by the National Institute for Health and Care Research (NIHR) as “doing research ‘with’ or ‘by’ people who use services rather than ‘to’, ‘about’ or ‘for’ them” [28]. This is a growing field, and as such there are several definitions and practices for PPI, but these generally centre around the active and transformative involvement of ‘lay representatives’ who contribute distinct perspectives to those of health professionals, due to their lived experience [29, 30].

Despite this increased recognition of the benefits of PPI, young people are often excluded from the process of influencing mental health research, and their involvement remains understudied [13–15, 30]. While efforts have been made to outline the benefits of youth involvement, and to develop practical recommendations for involvement [3], PPI with young people can remain complicated and ‘messy’ but success is achievable [31] and remains important for the future of youth mental health research.

### Responsible research and innovation (RRI)

RRI meanwhile has emerged more recently in the early 21st century, and is rooted in science and technology studies [32]. Since its inception, RRI has been integrated into numerous initiatives, most notably within the European Union’s Framework Programmes, particularly the Horizon 2020 Science with and for Society programmes [33]. In the UK, RRI has gained prominence through the UK’s Research and Innovation (UKRI) funding body, which encourages researchers to adopt the AREA framework as a guide for responsible practice [32].

Despite its growing relevance, RRI remains a concept with varied interpretations, and the definition of RRI remains diverse and lacks consensus [8, 34, 35]. For the purposes of this scoping review, we understand RRI as a broad approach for governing research and innovation responsibly across its lifecycle. It seeks to foster creativity while anticipating potential risks and unintended consequences, encouraging researchers and professionals to reflect on their assumptions and practices, engage relevant stakeholders, and respond to emerging knowledge, concerns, and societal needs. RRI therefore emphasises alignment with societal values and the public interest, while also incorporating the perspectives and needs of relevant stakeholders, including end users, throughout the research process [32, 35–37]. Hence, the relevance of RRI to this study lies in its potential to contribute to settings involving sensitive lived experience, fluctuating wellbeing, safeguarding and privacy concerns, youth-adult power asymmetries and digital innovations with uncertain or unintended consequences, such as youth digital mental health research [8, 11, 38, 39]. By foregrounding anticipation, inclusion, reflexivity, responsiveness and accountability, RRI complements PPI by situating involvement within a broader, lifecycle-oriented governance approach.

### Understanding PPI and RRI in youth mental health research

While these two approaches share some commonalities, they differ in scope and emphasis [8, 26, 30]. PPI is mainly concerned with research conducted with or by patients and members of the public, rather than to them, about them or for them, and foregrounds lived and experiential knowledge within health research [26, 28, 29, 40, 41]. RRI, by contrast, emphasises anticipating potential benefits, risks and unintended consequences; reflecting on assumptions, values and power relations [32, 35–37, 42]. Therefore, RRI does not replace PPI but can complement it by situating involvement within a wider approach of anticipation, reflexivity, responsiveness and accountability [8, 11, 38, 39]. For example, PPI may feasibly provide one mechanism through which the engagement dimension of RRI is enacted as part of the lifecycle for the wider research project.

Within youth mental health research, emerging scholarship further demonstrates the suitability of RRI. For instance, RRI-informed work has shown how youth involvement can be integrated with the anticipation and mitigation of harms in the development of digital interventions addressing self-harm [39], how responsible co-production requires care, emotional safety, dialogue, flexibility and power-sharing [11], and how RRI can be operationalised through youth leadership, intergenerational dialogue, safeguarding-sensitive practices and continuous reflexivity [8]. It has also translated RRI principles into practical guidance for meaningful, protective, inclusive and accountable youth involvement across mental health research and innovation work [38].

In practice, the literature tends to apply the labels ‘PPI’ and ‘RRI’, as well as related terms, to activities ranging from brief consultation, such as commenting on a participant information sheet or draft survey, to sustained, co-governed partnerships in which young people shaped research questions and/or a study’s methods and the proposed dissemination efforts. This breadth of interpretation spans disciplines and sectors, with some projects restricting PPI to service-improvement activities and others using RRI to support a broader, more systemic and integrated approach throughout the research process, which entails liaising with different layers of stakeholders while keeping young people’s well-being and views at the centre of understanding, prioritisation and decision-making [8, 11, 39].

This variation in definitions and operationalisation is further complicated by terms such as “co-design” and “co-production” being used interchangeably, although these approaches may be incorporated within PPI or RRI without being synonymous with either approach [8, 22, 26, 38]. Their use alone does not demonstrate that young people are involved as partners or that PPI or RRI was rigorously applied [8, 9, 21, 22, 26, 31]. Unlike broader research ethics frameworks that primarily focus on procedural compliance, risk mitigation, and participant protection, both PPI and RRI also emphasise relational and processual dimensions of research. For instance, PPI foregrounds lived experience and democratic involvement in health research [26, 28, 29, 40, 41], whereas RRI more explicitly incorporates anticipation, reflexivity, responsiveness, and societal alignment across the research lifecycle [8, 32, 35–37, 42]. This complexity may lead to research deploying these different approaches without appropriately considering their distinctions, theoretical foundations, and practical requirements, thereby blurring differences in the aims, depth of involvement, and responsibilities attributed to young people, as well as compromising transparency, comparability and possible efficacy [21–23, 30]. Hence, there is a need for clearer articulation of how PPI and RRI are understood and applied in the youth mental health field, so that researchers can more deliberately select, implement, and report approaches that are appropriate to their goals and to the forms of youth involvement envisaged. In this sense, clearly defining and distinguishing these principles within mental health research is crucial for improving how they are applied and, ultimately, for enabling the meaningful involvement of young people in research processes.

Involving young people in mental health research can enhance the relevance and appropriateness of research agendas, questions, and design by ensuring they are youth-friendly and responsive to young people’s needs, perspectives, and developmental stages [3, 4, 8, 11, 13–19, 43]. Meaningful involvement can also support implementation, interpretation, and dissemination efforts, while strengthening the contextual and cultural responsiveness, acceptability, and potential benefits of research [4, 12, 14, 16–20, 39, 44]. These benefits extend to the development of mental health products and tools, such as digital interventions, which may increasingly value the input of young people [18, 39, 45–47]. The importance of involving young people in mental health research has been recognised across diverse settings [9, 14, 20, 39, 48, 49]. However, a clear understanding of how projects are implementing youth meaningful involvement in line with PPI and RRI principles is needed [8, 9, 26, 31, 38]. This would help maximise the impact of young people’s contributions and support mental health researchers and professionals in making well-informed decisions about how to effectively design and conduct such projects [30].

Several previously published reviews have examined related dimensions of youth involvement in mental health research, although their scopes have been more circumscribed. McCabe et al. [14] synthesised the impacts, challenges, facilitators and recommendations associated with what the authors called ‘youth engagement’, whereas Ali et al. [50] focused specifically on young people with lived experience involved in the design, development or evaluation of mental health-care services. Totzeck et al. [51] reviewed PPI involving children and young people, identifying variation in how PPI processes were implemented and reported and calling for clearer operationalisation and evaluation, while Perowne et al. [52] subsequently concentrated on the barriers and facilitators affecting the involvement of under-represented groups. Other reviews have focused on online contexts and have addressed specific digital domains, including preventive digital interventions, digital-video interventions for mental health literacy, and the ethical promises and challenges of digital mental health, with co-design or end-user involvement examined as one component of broader intervention- or ethics-focused analyses (e.g., Bergin et al., 2020; Ito-Jaeger et al., 2022; Wies et al., 2021). Collectively, these reviews have established the value of involving young people and expose persistent weaknesses in implementation, evaluation and reporting; however, they do not comparatively examine how PPI and RRI are conceptualised and operationalised across the broader range of youth mental health-related research, intervention and product-development projects, nor have they systematically mapped the reported types of PPI and RRI, or the extent of young people’s involvement within these.

Within this context, the purpose of the present scoping review was to identify and map the existing literature on how collaborative approaches, specifically PPI and RRI, have been conceptualised and operationalised in mental health-related projects^2^involving young people. Given that PPI and RRI are inherently context-dependent, yet often employed interchangeably in collaborative initiatives [2, 8, 31], a nuanced understanding of how these approaches function in practice is essential. Moreover, there is a pressing need for a deeper comprehension of youth-inclusive collaborative processes in the mental health domain [8, 11, 30].

This review focused on studies that report on mental health-related research, interventions, or product development involving young people (10 to 25 years old) as collaborators, rather than solely as research participants. The central concepts under review are the PPI and RRI approaches as they apply to young people’s involvement in mental health research contexts. The context is defined as any mental health-related project (whether clinical, community-based, or digital) that explicitly incorporates collaborative involvement of young people beyond their participation as study subjects. The following research questions guided this review:

1. How does the existing literature define the PPI and RRI approaches in mental health-related projects involving young people?
2. What is the type and extent of young people’s involvement in PPI and RRI mental health-related projects where these are used?

These questions were designed to elucidate definitional clarity, implementation patterns, and levels of youth involvement across varying settings and methodologies, thereby informing future practice and research on collaborative youth involvement in mental health.

## Method

This scoping review was conducted following the JBI methodology for scoping reviews and reported following the PRISMA-ScR guidelines [53, 54]. The review protocol was registered on the Open Science Framework (DOI: https://doi.org/10.17605/OSF.IO/N4EDB) and has been published [30]. The review was not limited to a specific setting and encompassed diverse research contexts, provided they involved young people in mental health-related research or intervention development.

### Eligibility criteria

Eligibility criteria were established using the PCCS framework (Participants, Concept, Context, and Sources) [54], as follows: (Table 1) 

**Table 1:** PCCS acronym and inclusion and exclusion criteria

CATEGORY	CRITERIA	
	Inclusion	Exclusion
*Participant*	Articles describing and/or evaluating mental health-related research processes and product or intervention development in collaboration with young people. We considered ‘young people’ in a broad sense – ranging from the early stages of adolescence (i.e., 10 years old) to early adulthood (i.e., until 25 years old – WHO, 2024) [73]	Articles focusing on another population rather than on young people (as operationalised in the ‘inclusion’ criterion)
*Concept*	Articles that explore and discuss the concepts and the processes of the ‘patient and public involvement’ (PPI) and/or ‘responsible research and innovation’ (RRI) approaches. These include, but are not limited to, face-to-face, online and hybrid mental health-related research processes and product or intervention development in collaboration with young people	Articles not focusing on PPI or RRI by just mentioning them *en passant*
*Context*	Articles reporting mental health-related research processes and product or intervention development in collaboration with young people	Articles not focusing on mental health-related research or product development processes
*Sources* *(Type of studies)*	We included only studies using primary data that were peer-reviewed and published in English with no time restriction – i.e., we retrieved databases’ articles since their inception. We included quantitative, qualitative, and mixed methods studies	The review excluded prospective study protocols, review articles, and general ‘grey literature’

### Search strategy and information sources

A two-step search strategy was employed. Initially, a limited search was conducted in PsycARTICLES, PsycINFO, and MEDLINE to identify relevant literature. In particular, the titles, abstracts, author-supplied keywords and database indexing terms of potentially relevant records were examined to identify alternative terminology relating to young people, mental health, PPI, RRI and associated forms of collaborative involvement. These terms were then used to further refine and then expand the comprehensive search strategy, which was subsequently adapted to the controlled vocabulary, syntax and indexing conventions of each database. No substantive changes were made to the review questions, PCCS eligibility criteria or the conceptual scope between the two steps. Hence, the changes concerned the refinement and then expansion of search terms and their database-specific adaptation. The refined search strategy was subsequently applied to MEDLINE, PsycINFO, PsycARTICLES, Scopus, Web of Science, IBSS, CINAHL (EBSCO), and ASSIA. The final search strategy, including all descriptors and search strings, is available in Additional File 1.

### Study selection

All identified records were collated and uploaded into CADIMA, an open-access platform designed to support the conduct and documentation of systematic and scoping reviews [55]. CADIMA was used to manage part of the selection process, including de-duplication, consistency checking, and inter-rater reliability testing. A total of 2,638 duplicates were removed from the initial 3,806 records.

Prior to the title and abstract screening phase, a consistency check was conducted on a randomised 5% sample of the remaining records. This process, supported by CADIMA’s automated inter-rater reliability function, yielded a Cohen’s Kappa coefficient [56] of 0.86, indicating ‘excellent agreement’ between the first and second authors. The two reviewers then independently screened 1,168 titles and abstracts in parallel. Any disagreements regarding study selection and data extraction were resolved through consensus and, when necessary, further discussion. A total of 124 articles were subsequently selected for full-text review.

**Fig. 1:**
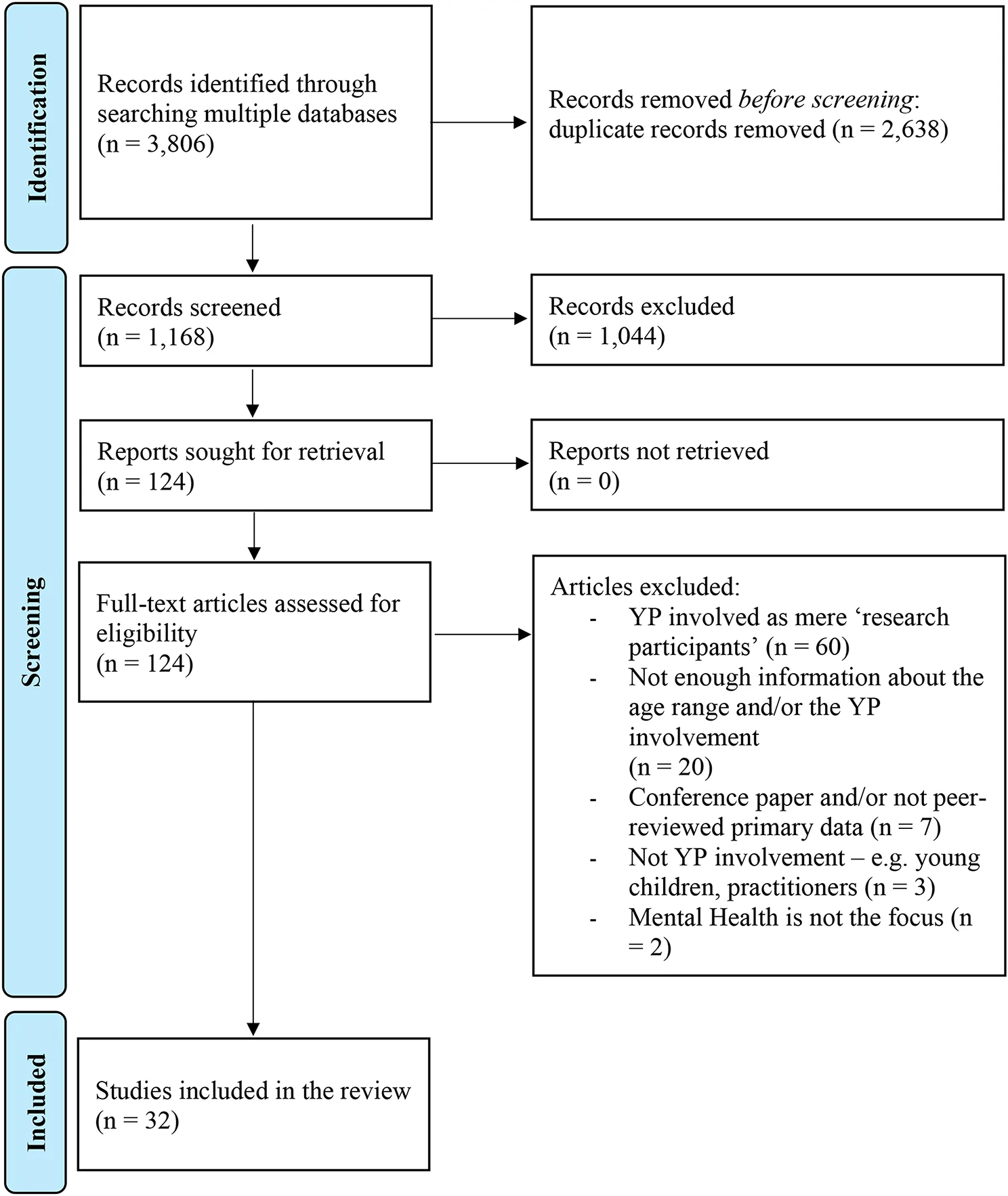
Review process

Although the original protocol specified that full-text screening would be conducted by two reviewers, due to project constraints (e.g., available personnel and funding deadlines in relation to staffing), this task was carried out solely by the first author. However, to ensure rigour, the third author reviewed all exclusion decisions and confirmed agreement. The list of excluded full-text articles, along with reasons for exclusion, is provided in Additional File 2. The study selection process is summarised in the PRISMA-ScR flow diagram (Fig. 1).

### Critical appraisal

No formal quality or risk-of-bias appraisal of the included studies was undertaken. This is consistent with established scoping-review guidance, as such appraisals are not considered necessary, because the review aimed to map and clarify how PPI and RRI were conceptualised and operationalised and to characterise the reported types and extent of youth involvement, rather than to evaluate intervention effectiveness or produce a critically appraised answer to a narrowly defined question [53, 54, 57].

### Data extraction and analysis

Data was extracted from the 32 studies included in the review using a structured data extraction tool developed by the authors. This tool was designed to capture comprehensive and consistent information aligned with the objectives and research questions of the review. The extracted data were grouped into four analytical categories:

1. **General descriptions and methods**: key study characteristics, including the collaborative approach adopted (PPI, RRI, other, or unclear), the number of young people involved, their age range, mean age (when available), and the study’s methodological design (qualitative, quantitative, mixed methods, or other). Operational definitions are provided in Table 2;
2. **Involvement characterisation**: recorded the specific mental health topic addressed, the reported form or role of young people’s involvement (e.g., Co-researchers/Co-developers, Co-design, Consultation, other), the mode of collaboration (online, in person, hybrid, or unclear), and whether young people were listed as co-authors. The associated operational definitions are outlined in Table 3. These categories were used as pragmatic descriptors of how involvement was reported and were not treated as direct measures of its depth, quality or distribution of decision-making power. The terminology adopted by a study was therefore considered separately from the activities undertaken, the stages at which young people were involved and their reported influence over project decisions;
3. **Definitions of involvement**: whether the study provided explicit definitions of PPI and/or RRI, noting the presence or absence of definitional clarity in relation to the collaborative approaches adopted;
4. **Complementary information**: captured additional insights, including whether the article discussed the main advantages and/or challenges of collaborating with young people, and whether it presented examples of good practices for fostering meaningful and ethical collaboration.

**Table 2:** Operational definitions for the ‘general descriptions and methods’ category

Concept	Key Definition	Source
*PPI (Patient and Public Involvement)*	Patient and Public Involvement (PPI) is the active involvement of patients and the public in research, where research is conducted *with* or *by* members of the public, rather than merely *to*, *about*, or *for* them, promoting collaboration and mutual engagement^**a, b**^. This inclusive approach enhances the quality, relevance, and application of research, as it brings in the unique perspectives and lived experiences of service users, leading to more insightful prioritisation, commissioning, and dissemination of research findings^**b**^. Recognising the diversity of roles within PPI, terms such as “PPI representatives” and “lay representatives” are often used interchangeably, reflecting the broad, non-professional insights contributed by these individuals, whether as patients, carers, or members of the public^**c**^. PPI spans multiple levels of involvement, employing methods such as communication, consultation, and involvement across different stages of research and guideline development, from topic selection to implementation in clinical and community settings^**d**^.	**a.** Papageorgiou et al. (2023) [62] **b.** Brett et al. (2014) [16] **c.** Wilson et al. (2015) [29] **d.** Boivin et al. (2010) [74]
*RRI* *(Responsible Research and Innovation)*	Responsible Research and Innovation (RRI) is a process that promotes the development of research and innovation that is socially desirable, ethically acceptable, and aligned with the values, needs, and expectations of society^**a, b**^. It encourages inclusiveness, transparency, and responsiveness, aiming to engage societal actors and researchers collaboratively, fostering a more democratic accountability across the entire innovation lifecycle^**c, d, e**^. RRI operates as an anticipatory, reflexive, and deliberative approach, where ethical and social implications are assessed from the initial design stages through to application, prioritising public interest and minimising harm^**f, g**^. Additionally, RRI recognises that responsibility is a distributed network across stakeholders, including policymakers, scientists, and the public, to achieve sustainable, innovative solutions that address significant societal challenges^**a, d**^.	**a.** Inglesant et al. (2016) [75] **b.** Eden et al. (2014) [42] **c.** Shahriari and [42] **c.** Shahriari and Shahriari (2017) [34] **d.** Hoven (2021) [76] **e.** Porcari et al. (2016) [77] **f.** Von Schomberg (2013) [37] **g.** Owen and Pansera (2019) [35]

**Table 3:** Operational definitions for the ‘involvement characterisation’ category

Concept	Key Definition	Source
*Co-researchers/* *Co-developers*	In the ‘Co-researchers/Co-developers’ model of youth involvement, young people are involved as integral members of the research or development team, contributing their expertise as “lived experience” experts rather than as traditional study subjects^**a, b**^. This role involves young people working alongside academics and/or professionals, actively engaging in all project stages (e.g., conception, planning, execution, delivery and dissemination) where they are fully embedded in decision-making processes^**c**^. In this capacity, young people collaborate to shape the project’s direction and outcomes, ensuring their insights and experiences are foundational to the project’s development and its relevance to target communities^**d, e**^.	**a.** Fraser et al. (2022) [78] **b.** Martin et al. (2019) [10] **c.** Pavarini et al. (2019) [72] **d.** Hawke et al. (2018) [3] **e.** McPartlan (2024) [79]
*Co-design*	Co-design is a collaborative design process rooted in participatory design that actively involves users, stakeholders, and designers as partners throughout all stages of development, incorporating their lived experiences and unique insights to create solutions that are relevant and responsive to users’ needs and preferences^**a, b, c**^. This approach values users as “experts of their experiences” and employs participatory methods like workshops and interactive activities to ensure design outcomes are informed by real-life perspectives and are both ethical and practical^**a, c**^. Beyond traditional consultation, co-design empowers users in decision-making and draws on multidisciplinary insights from social policy, product design, and public sector reform to address complex social needs^**d, e**^.	**a.** Thabrew et al. (2018) [45] **b.** Bevan Jones et al. 2020) [46] **c.** Malloy et al. (2023) [47] **d.** McKercher (2020) [80] **e.** Evans and Terrey (2016) [81]
*Consultation*	Young People Consultation involves gathering young people’s views to inform the development or revision of projects, services, or research, typically by seeking their opinions on materials, processes, or policy decisions^**a, b**^. Consultation is generally distinct from full involvement, as it is often limited to asking for input rather than involving young people directly in decision-making^**b**^. This approach values young people’s unique perspectives, aiming to make resources more accessible and relevant by incorporating age-appropriate feedback, while also occasionally piloting their suggestions in practical applications to improve real-world services^**a, d**^.	**a.** Delahunt et al. (2023) [82] **b.** Brady (2020) [83] **c.** Sutterlüty and Tisdall (2009) [84] **d.** Woolfson et al. (2008) [85]

**Note:** the term ‘co-production’ was not used as an operational definition in this review, as the literature often employs it as an umbrella term encompassing a wide range of involvement practices, including ‘co-research’, ‘co-development’, ‘co-design’, and ‘consultation’, which were treated as distinct categories for the purposes of this study

For further details about the data extraction tool, see Additional File 3. All data were extracted by the first author and reviewed for consistency and completeness by the second author. Patterns and themes across the extracted data were subsequently analysed through descriptive synthesis, guided by the review questions and the emergent features of the included studies. For the subset of data focusing on definitions of PPI, RRI and related involvement approaches, we conducted a qualitative synthesis of these concepts. To support this conceptual synthesis, we used ChatGPT-4o as an assistive tool. The first author provided the model with sets of verbatim excerpts grouped by concept (see Additional File 4) and requested concise draft definitions based solely on those excerpts, which were iteratively refined. All AI-generated text was then reviewed to ensure fidelity to the source material (see Additional File 5 for further details of this process). Recent methodological work has shown that tools such as ChatGPT can assist with coding, clustering and summarising qualitative text, provided that researchers remain responsible for guiding the analysis and making final interpretative decisions [57–59]. Consistent with this human–AI collaborative stance, ChatGPT-4o was used only as an auxiliary aid to generate preliminary, text-bound syntheses.

### Young people’s involvement in this study

The authors AA and AM are young people with lived experience of mental health, and they are members of Digital Youth’s Young People’s Advisory Group (https://digitalyouth.ac.uk/), the programme that hosted this study. AA was actively involved in the conception and design of the study, data collection, as well as the revision of the manuscript. AA and AM also championed the development of a youth-accessible version of the results in the form of a voice-over video (https://bit.ly/3LQMG1w), for which they were supported by LR. AM is one of the wider programme’s Youth Co-Chairs^3^.This iterative and dialogic process with young people reflects Digital Youth’s commitment to embedding young people’s voices at multiple stages of the research across the entire research lifecycle (i.e., embedded rather than episodic), in alignment with core RRI values such as inclusivity, responsiveness and reflexivity. For more details, please see Mendes et al. [11].

### The impact of youth involvement in this review

The impact of young people’s involvement in this review can be understood across two interrelated domains: academic and research improvements, and the skill development and empowerment of young people. As described above, young co-authors contributed to the conception and design of the review, its data collection, and revision of the manuscript, and the creation of an accessible voice-over video presentation of the findings for a non-academic audience. Their involvement enabled young people’s perspectives to inform both the research processes and the ways in which its findings were interpreted, communicated and made accessible.

In relation to academic and research improvement, this review was designed and conducted during the early stages of the aforementioned broader research programme within which it was situated. Its preliminary findings, and the continuing dialogue between academic researchers and young people, helped identify conceptual and practical questions requiring further investigation, particularly concerning how RRI was understood and enacted within youth mental health research. This informed the design and delivery of a subsequent empirical study involving academic researchers and young people, which explored understandings and practices of RRI within the broader research programme [8]. Together, the present review and that empirical study provided key conceptual and experiential foundations for the development of the RISE-UP Framework [38], a practice-oriented framework that translates RRI into principles and reflective guidance for supporting meaningful, safe, inclusive and accountable youth involvement across youth mental health research.

Young people’s involvement in this review also supported their skill development and empowerment. Their contributions provided opportunities to develop knowledge and practical skills relating to literature scoping-review processes, academic writing, collaborative decision-making and research dissemination. Importantly, this enabled them not only to communicate their perspectives, but also to make decisions and exercise ownership over the work they had helped produce, in addition to how the work was represented publicly to a range of stakeholders. For example, one of the young people (author AM) was involved across this review and other related research activities, and they subsequently submitted an oral presentation to a major youth mental health conference and are scheduled to present the collaborative process through which the review, the completed studies and the RISE-UP Framework were developed. This youth co-author’s progression illustrates how sustained involvement can extend beyond contributions to an individual review or study by enabling young people to develop confidence, claim intellectual recognition and represent collaborative research in academic and other settings.

## Results

This section begins by outlining the general characteristics and frequency patterns of the included studies, offering an overview of the sample. Following this, the findings concerning each of the review questions are addressed, highlighting key trends and emerging patterns regarding how PPI and RRI have been conceptualised and implemented in mental health-related projects involving young people.

### Overview of study characteristics and distributions

From the earliest papers to 2024, a notable concentration of studies was observed from 2018 onwards, with a particularly marked increase in publications in the most recent years. The initial search yielded a total of 3,806 records, distributed across eight bibliographic databases. The largest number of records was retrieved from Scopus (*n* = 981), followed by Web of Science (*n* = 808), MEDLINE (*n* = 847), PsycINFO (*n* = 469), and CINAHL (*n* = 421). Smaller numbers of records came from ASSIA (*n* = 203), PsycARTICLES (*n* = 27), and IBSS (*n* = 50). These results indicate that literature relevant to youth collaboration in mental health contexts is widely indexed across multidisciplinary, health-focused, and social science-oriented databases, reflecting the cross-cutting nature of the topic.

Regarding the geographical distribution of the 124 papers assessed during the full-text screening phase, most of the selected (S = 27) and non-selected (NS = 77) studies originated from high-income, English-speaking countries. The majority were concentrated in the United Kingdom (S = 14; NS = 32), Australia (S = 5; NS = 21), Canada (S = 4; NS = 16), and the United States (S = 4; NS = 8), indicating a strong dominance of Global North contexts in the literature addressing collaborative approaches such as PPI and RRI in youth mental health research. In contrast, representation from regions of the Global South remained notably low. Only a small number of papers originated from Africa (S = 1; NS = 3), Asia or South Asia (S = 0; NS = 6), and South America (S = 2; NS = 1), highlighting a potential geographic gap in the academic discourse and the need for broader inclusion of diverse socio-cultural and regional perspectives in the field.

**Table 4:** General characteristics of the included studies

Paper ID	Mental Health Topic	Authors	Year	Country	Data Collection for PPI and RRI Approaches	Category of Search String	How was the collaboration held?	Number applicable YP	YP Age Range	YP Mean Age	YP Involvement	YP Coauthor?^a^
P1	Clinical eHealth Platform for Youth	Whitehouse et al. [86]	2013	Canada	Qualitative (interviews/focus groups with young people and professionals)	PPI	In person	80	12-18	N/A	Consultation	No
P2	Quality standards for youth mental health in primary care	Graham et al. [87]	2014	UK	Qualitative (interviews/focus groups with young people)	RRI	In person	50	16-25	N/A	Co-researchers/ Co-developers	No^1^
P3	Young people’s counselling service	Perry and Carpenter [88]	2016	UK	Qualitative (interviews/focus groups with young people)	RRI	In person	8	10-17	N/A	Co-design	No^1^
P4	Transition programme for youth leaving CAMHS	Dunn [89]	2017	UK	Qualitative (interviews/focus groups with young people)	PPI and RRI	In person	18	17-22	*M* =18.75	Co-design	No^2^
P5	Culturally adapting a web tool for YP clinical support	Ospina-Pinillos et al. [90]	2018	Australia	Mixed methods	RRI	In person	28	16-25	N/A	Co-design	No^2^
P6	Culturally adapting a web tool for YP clinical support	Ospina-Pinillos et al. [91]	2019	Australia	Mixed methods	RRI	In person	17	17-29; 19-30	*M* = 24 *M* = 26	Co-design	No^2^
P7	Using technology to detect mental health deterioration	Dewa et al. [92]	2021	UK	Qualitative (interviews/focus groups with young people and professionals)	PPI	Hybrid (both online/ virtually and in person)	7	18-25	N/A	Co-researchers/ Co-developers	Yes (listed individually)
P8	Benefits and risks of adolescents’ electronic image-sharing	Cardoso et al. [93]	2020	Australia	Qualitative (interviews/focus groups with young people)	PPI	In person	68	13-15	N/A	Co-design	No^2^
P9	Web-based mental health clinic for young people	Ospina-Pinillos et al. [48]	2020	Colombia	Qualitative (interviews/focus groups with young people and professionals)	RRI	Hybrid (both online/ virtually and in person)	12	18-22; 17-24	*M* = 19.5; *M* = 22	Co-design	No^2^
P10	App for youth with substance use and mental health challenges	Adams et al. [94]	2021	USA	Mixed methods	RRI	Hybrid (both online/ virtually and in person)	20	14-17	Phase 1: *M* = 15.3, *SD* = 1.0; Phase 2: *M* = 15.9, *SD* = 1.0	Consultation	No
P11	Help-seeking and mental health in abused and neglected CYP	Bennett et al. [95]	2023	UK	Qualitative (documental)	PPI	Hybrid (both online/ virtually and in person)	10	14-18	N/A	Co-researchers/ Co-developers	Yes (listed as a YPAG/ CYPAG)
P12	Help-seeking and mental health in abused and neglected CYP	Bennett et al. [96]	2022	UK	Qualitative (documental)	PPI	Hybrid (both online/ virtually and in person)	10	14-18	N/A	Co-researchers/ Co-developers	Yes (listed individually)
P13	Help-seeking and mental health in abused and neglected CYP	Bennett et al. [97]	2022	UK	Qualitative (documental)	PPI	Hybrid (both online/ virtually and in person)	10	14-18	N/A	Co-researchers/ Co-developers	Yes (listed individually)
P14	Indigenous youths’ experiences accessing mental health care	Sam et al. [98]	2022	Canada	Qualitative (interviews/focus groups with young people)	PPI	In person	8	20-24	*M* = 22	Co-design	Yes (listed as a YPAG/ CYPAG)
P15	Youth public mental health	Taylor et al. [99]	2022	UK	Qualitative (interviews/focus groups with young people)	PPI	In person	16	16-24	N/A	Consultation	No^1^
P16	Digital mental health for vulnerable youth in UK lockdown	Mindel et al. [60]	2022	UK	Qualitative (interviews/focus groups with young people and professionals)	PPI	Online/ Virtually	11	16-25	N/A	Co-design	No^1^
P17	Emotion regulation app (Eda) for children	Moltrecht et al. [100]	2022	UK	Qualitative (interviews/focus groups with young people and professionals)	PPI	In person	33	12-19	N/A	Co-design	No^1^
P18	CBT app for adolescent depression and anxiety	Li et al. [101]	2022	Australia	Qualitative (interviews/focus groups with young people and professionals)	PPI	Online/ Virtually	36	12-16	*M* =14.94, *SD* =1.3	Co-design	No
P19	Safeguarding mental resilience amid young adult drug use	Ben-Yehuda et al. [102]	2022	Israel	Mixed methods	RRI	In person	339	18-30	*M* = 24.3	Consultation	No
P20	Transition readiness in youth with chronic and mental health conditions	Allemang et al. [103]	2023	Canada	Mixed methods	PPI	Online/ Virtually	5	18-30	N/A	Co-researchers/ Co-developers	Yes (listed individually)
P21	Culturally adapting self-guided youth mental health interventions	Shroff et al. [104]	2023	USA	Mixed methods	PPI	Online/ Virtually	14^3^	12-17	N/A	Co-design	No
P22	Arts-based methods in online youth mental health research	Woodgate et al. [105]	2023	Canada	Qualitative (interviews/focus groups with young people)	PPI	Online/ Virtually	7	19-28	N/A	Co-design	No^1^
P23	Digital suicide prevention for LGBTQ+ youth facing online victimisation	Biernesser et al. [106]	2023	USA	Mixed methods	PPI	Online/ Virtually	33	13-18	*M* = 15.7	Co-design	No^1^
P24	Gamified chat-story for youth mental health in Brazilian schools	Siston et al. [4]	2023	Brazil	Qualitative (descriptive, e.g., ethnography – young people and researchers)	PPI	Hybrid (both online/ virtually and in person)	5	17-20	N/A	Co-researchers/ Co-developers	Yes (listed individually)
P25	Adolescent mental health in post-primary settings	Neill et al. [107]	2023	UK	Qualitative (interviews/focus groups with young people and professionals)	PPI	In person	32	12-18	N/A	Co-design	No^1^
P26	Acceptability of brief CBT for youth with OCD	Waite et al. [108]	2023	UK	Qualitative (interviews/focus groups with young people)	PPI	Online/ Virtually	5	12-22	N/A	Consultation	No^1^
P27	Digital mental health for anxiety, depression, and related issues	Ludlow et al. [109]	2023	Australia	Qualitative (interviews/focus groups with young people)	PPI	In person	68	13-16	*M* = 14.57, *SD* = 1.89	Co-design	No
P28	Co-creating web resources for mental health literacy	Ito-Jaeger et al. [19]nterviews/focus groups with young people)	PPI	Online/ Virtually	30	17-21	*M* = 19, *SD* = 1.509	Co-design	No			
P29	Emerging mental health problems	van Doorn et al. [110]	2023	The Netherlands	Mixed methods	RRI	Online/ Virtually	26	18-25	*M* = 22,3, *SD* = 2,15	Consultation	No
P30	Youth experiences of psychosis during COVID-19 in Nigeria	Bella-Awusah et al. [49]	2024	Nigeria, Africa	Qualitative (interviews/focus groups with young people)	PPI	In person	20	18-25	N/A	Co-researchers/ Co-developers	Yes (listed individually)
P31	Mental health views of youth with complex needs	Sims-Schouten et al. [111]	2024	UK	Qualitative (interviews/focus groups with young people and professionals)	PPI	Hybrid (both online/virtually and in person)	14	12-14	N/A	Co-design	No
P32	Mental health, COVID-19 and filmmaking	Baumann et al. [61]	2024	USA	Qualitative (arts-based – young people)	RRI	In person	8	15-19	N/A	Co-design	No

References: CAMHS = Child and Adolescent Mental Health Services; CYP = Child and Young People; CBT = Cognitive Behavioural Therapy; LGBTQ+ = lesbian, gay, bisexual, transgender/trans; YP = Young People

^a^Paper to explicitly state YP was a co-author drawing on their lived experience

^1^Young people (as experts by experience) explicitly mentioned in the acknowledgements, but not by name

^2^Young people (as experts by experience) mentioned explicitly in the acknowledgements, but not by name. Plus, certain people are cited by name in the acknowledgments, but it is unclear if they are young people with lived experience

^3^Young people involved in the consultation, co-design or co-research/co-development, separate from the overall total number of young people involved in evaluating the associated intervention

Table 4 summarises the general characteristics of the studies included in this review. Although no date restrictions were applied, no eligible studies published before 2013 were identified. This absence may reflect the later institutionalisation of PPI, RRI and related involvement-oriented approaches in youth mental health, as well as changes over time in the terminology used to describe collaborative roles. Earlier relevant work, especially regarding RRI, may also have been indexed under different terms or may not have been adequately captured by contemporary database vocabularies. This review cannot determine whether this pattern reflects a genuine absence, terminology and indexing effects, or a combination of these factors; nevertheless, it is consistent with the subsequent growth of the field.

Only 11 (34%) of the included studies provided the mean age of youth involved, and just 5 (16%) reported the standard deviations, highlighting ongoing limitations in the consistent reporting of demographic data in youth-involved research. In terms of methodological design, qualitative approaches predominated. Specifically, 12 studies (38%) conducted qualitative studies exclusively with young people, whereas 9 studies (28%) included both young people and professionals/researchers. Eight (25%) studies adopted mixed-methods designs, while others employed documentary research methods (e.g., social media and online forum posts) (*n* = 3; 9%).

For the search strategies, the majority of included articles (*n* = 22; 69%) were retrieved through the PPI search string, while 9 (28%) were identified via the RRI search string, and only one (3%) was retrieved by both – see Additional File 1 to check the search strings. This distribution reflects the comparatively greater operationalisation of PPI approaches in youth mental health research. Regarding the modality of collaboration, nearly half of the studies (*n* = 15; 47%) reported in-person involvement, while 9 (28%) were conducted exclusively online or virtually, and 8 (25%) employed hybrid formats that combined both.

When examining the type and extent of involvement, most studies involved young people in co-design processes (*n* = 18; 56%), followed by more integrated roles as co-researchers or co-developers (*n* = 8; 25%), and less extensive forms of consultation (*n* = 6; 19%). Notably, 8 (25%) studies listed young people as co-authors – 6 (19%) of these naming individuals and 2 (6%) attributing authorship to a youth advisory group (e.g., Young Person’s Advisory Group/YPAG). However, 1 (3%) study that positioned young people as co-researchers or co-developers did not appear to list them as co-authors, raising potential concerns about the consistency between the nature of their involvement and recognition of their contributions.

### Conceptualisations of PPI and RRI approaches and levels of youth involvement

One of the primary aims of this scoping review was to examine how youth mental health projects and initiatives define and apply the conceptual approaches of PPI and RRI. Despite the centrality of these approaches to collaborative research, our findings reveal a relatively limited articulation of either across the 32 included studies.

Only six papers explicitly referred to PPI by name (P7, P12, P16, P17, P24, P26), with just two of these offering an operational definition (P7, P26). Perhaps unsurprisingly five out of the six papers were from the UK, given PPI is the terminology employed by key research funders in the UK (e.g., NIHR) – P24 was conducted in Brazil but with funding and a principal investigator from the UK. Here is a synthesised definition which has drawn upon two of the six papers (the only ones to offer a definition):

PPI is defined as research conducted with or by patients and the public, rather than to, for, or about them (Paper/P7, P26). It is a dynamic, context-specific interaction shaped by multiple factors (P7). Recent shifts in research infrastructure and increased patient voice have promoted more meaningful involvement and co-production, moving away from tokenism. Funders and journals increasingly require researchers to report how patients, carers, or the public are involved, leading to the creation of institutional PPI resources (P7). PPI activities such as co-design and co-production improve the acceptability of research interventions and processes (P26)

In contrast, the RRI approach was scarcely cited. Only one paper (P28) mentioned RRI explicitly, and even then, without offering an operational definition.

**Table 5:** Operational definitions for the ‘definitions of involvement’ category

	Patient and Public Involvement		Responsible Research and Innovation	
*Provide a definition*	Mentions PPI and/or uses related terms (e.g., service user involvement, patient-oriented, end-user involvement, co-production, co-design, co-creation, co-operative, single-session intervention) and cite references	P1, P2, P4, P7, P12, P15, P18, P21, P22, P23, P24, P26, P27, P31	Mentions RRI and/or uses related terms (e.g., participatory research, participatory design, peer research, UX (user experience), human-centred design, user-guided/centred design, agile development, stakeholder involvement/engagement) and cite references	P2, P4, P5, P9, P10, P22, P31
*Do not provide a definition*	Mentions PPI and/or uses related terms (e.g., service user involvement, patient-oriented, end-user involvement, co-production, co-design, co-creation, co-operative, single-session intervention) and cite references	P11, P13, P20, P25	Mentions RRI and/or uses related terms (e.g., participatory research, participatory design, peer research, UX (user experience), human-centred design, user-guided/centred design, agile development, stakeholder involvement/engagement) and cite references	P6, P19, P25, P28, P29
	Mentions PPI and/or uses related terms (e.g., service user involvement, patient-oriented, end-user involvement, co-production, co-design, co-creation, co-operative, single-session intervention) but do not cite references	P4, P6, P8, P12, P13, P14, P16, P17, P22, P23, P24, P26, P27, P28, P29, P30	Mentions RRI and/or uses related terms (e.g., participatory research, participatory design, peer research, UX (user experience), human-centred design, user-guided/centred design, agile development, stakeholder involvement/engagement) and do not cite references	P3, P10, P15, P17, P18, P22, P23, P24, P27, P31

As shown in Table 5, almost half of the included studies (*n* = 14; 44%) provided a definition and corresponding citations for PPI or conceptually related terms such as “service user involvement”, “patient-oriented”, “end-user involvement”, “co-production”, or “co-design”. Notably, two of these studies had been retrieved through RRI search strings, reflecting an intersection in terminological usage. An additional four studies (13%) mentioned PPI-related terms and cited relevant literature without offering definitions. However, 50% of the papers (*n* = 16) referenced PPI or associated terminology without providing any citations or definitions, including three studies initially retrieved via RRI search strings. Overall, four RRI papers (P2, P4, P6, P29) defined or discussed PPI-related concepts.

The definitional clarity for RRI was even more limited. Just seven studies (22%) provided a definition and citations for RRI or related terms (e.g., participatory research, user-centred design, stakeholder engagement), two of which originated from PPI search strings. Five studies (16%) mentioned RRI-related concepts and provided supporting citations, but no definition, while approximately one third of the papers (*n* = 10; 31%) only referenced such terms without definition or citation. Of those, eight had been retrieved via PPI search strings, again indicating overlapping use of language. In total, nine PPI papers (P15, P17, P18, P22, P23, P24, P27, P28, P31) explicitly addressed RRI-related terminology, further highlighting the conceptual blending and the need for more consistent operationalisation of both approaches in the literature.

This conflation between PPI and RRI is also evident in the way several studies mix terms from both approaches. For example, P17 combines references to both “user-centred” design and PPI; P18 draws on “human-computer interaction” and “UX (user experience)”; P22 incorporates “co-design”, “co-research”, and “co-creation” alongside “participatory research”; and P24 includes references to “co-production”, “peer research”, and “citizen science”. Although this blending of terminology might reflect the interdisciplinary and evolving nature of collaborative research with young people, it also complicates efforts to disentangle the theoretical underpinnings and practical distinctions between PPI and RRI.

It is also important to note that in both PPI and RRI papers, terms such as “co-design” were often used adjectivally or descriptively rather than as formal methodological approaches (P12, P16, P17, P18, P23, P24, P26). This suggests that, while collaborative language is widely adopted, the systematic application of involving methodologies remains inconsistent. Moreover, some RRI papers, for example, P10, did not present or define any approach of collaboration or involvement, further indicating the lack of conceptual clarity and operational precision within this body of literature.

Despite this, the relevance of RRI to youth mental health research is not absent. For instance, P24 acknowledges that

co-production, participatory research, peer research and citizen science are some of the terms used within a large body of emerging concepts and models that advocate public participation in research and require new skills and ways of producing and communicating knowledge (p. 1)

This statement reinforces the argument that RRI-aligned principles are implicitly present, even if the approach itself is not explicitly acknowledged or defined.

Collectively, the results indicate that PPI remains more frequently defined, cited, and operationalised within youth mental health projects, particularly those conducted in high-income countries. However, the vocabulary and values associated with RRI (particularly those emphasising ethics, inclusion, responsiveness, and reflexivity) clearly resonate across the included studies, pointing to a need for more integrated and transparent conceptual framing in future collaborative research with young people.

To further illustrate the diversity and depth of involvement approaches referenced across the included studies, Table 6 compiles definitions of collaborative approaches found in the sample. These definitions, derived directly from the reviewed literature, reflect the varying ways in which terms associated with both PPI and RRI, such as co-production, co-design, participatory research, participatory design, and design thinking, were conceptualised within youth mental health projects. In cases where multiple studies addressed the same concept, definitions were synthesised; where only one study engaged with a term, the original verbatim is presented. By presenting these definitions, Table 6 offers insight into how involving methodologies are framed across the field and supports comparative analysis of their scope, principles, and alignment with broader youth involvement approaches.

**Table 6:** Definitions for involvement approaches in youth mental health

Approach	Concept/Definition			Source
PPI	Arts-based Methods:“participatory in nature, arts-based methods (ABM) have been defined as “research methods in which art forms play a primary role in any or all of the steps of the research process” (p. 1)			P22
	Co-creation: “[this] process shifts away from the traditional method of involving passive stakeholders during the latter phase of prototype testing towards viewing them as active contributors with knowledge and skills for co-creation during the ideation phase” (pp. 3-4)			P1
	Co-design:it is a collaborative design approach that meaningfully engages end-users and stakeholders throughout the development process^P27^. It involves sharing knowledge about both process and content to reach a common goal^P22^, and requires active involvement, beyond consultation, through methods such as discussions, mapping, and prototyping^P27^. For young people, this means creating age-appropriate, relatable, and lifestyle-relevant interventions^P18^. In digital mental health, co-design is seen as best practice for adoption and equity, as it centres lived experience and considers cultural and technological contexts^P23^.			P18, P22, P23, P27
	Co-operative Enquiry:“this entailed working ‘with’ rather than ‘on’ young people. (…) Co-operative enquiry often explores ways of transforming processes and procedures (on personal and organisational levels) and identifying best practice though cycles of reflection and action” (p. 173)			P3
	Co-production:it is a research approach in which researchers, practitioners, and members of the public work together, sharing power and responsibility throughout all stages of the project^P24^. It values the diverse skills, experiences, and roles of all contributors, promoting more democratic and inclusive processes^P24, P31^. Originally developed in service settings, co-production recognises young people as active agents rather than passive recipients and has shown benefits in mental health research, including improved well-being^P15^. Its core principles include power sharing, reciprocity, inclusion, respect for all knowledge, and relationship building^P7, P12^.			P7, P12, P15, P24, P31
	Design Thinking: it is a participatory, iterative process that involves end-users in the co-creation and evaluation of digital tools and resources, such as e-mental health applications^P14^. It typically follows a Double-Diamond model comprising four phases: discovering and defining issues, developing and selecting potential interventions, and delivering prototyped solutions^P8^.			P4, P8
	Participatory Research:it is a democratic and collaborative approach rooted in community development that aims to redress power imbalances between researchers and participants^P4^. It treats research as a shared exercise, involving participants, such as patients, as co-researchers through reflexivity, disclosure, and mutual interpretation of findings^P2^. Often framed as cooperative enquiry, this model values diverse perspectives and promotes equal involvement ^P2^. PR may include creative, arts-based, and non-verbal methods, which are especially suitable for engaging vulnerable groups and exploring complex or sensitive issues^P4^.			P2, P4
	Single-session Intervention (SSI):“[it] may help address the need for accessible, easily completable, and effective mental health interventions for young people. [SSI is] defined as “structured programs that intentionally involve only one visit or encounter with a clinic, provider, or program; they may serve as stand-alone or adjunctive clinical services”. [SSI] can take multiple forms (eg, provider delivered and self-guided); however, the flexible and scalable nature of digital, self-administered SSIs allows them to be delivered and completed in low-resource settings relatively rapidly and broadly. Existing literature supports the effectiveness of SSIs for young people’s depression and anxiety, even when delivered as stand-alone mental health supports” (p. 2)			P21
RRI	Agile Development (System/Software):“agile system development – a set of practices in which requirements are gathered and the software is developed, tested, and improved in an iterative process”. (…) agile software development is a methodology in which requirements are gathered and the software is developed, tested, and further improved in an iterative process. The team that develops the software includes developers and potential users, an approach that is also known as participatory design (p. 4)			P19
	Community-based Participatory Research (CBPR):“participants are engaged throughout the research process, such as collecting data, coanalyzing the data with researchers, and making key decisions in sharing the research findings” (p. 370)			P32
	Discover, Design, Build, and Test (DDBT): “[it] provides a structure for DMHI development that features codesign with end-users. DDBT brings together principles of human centered design n (HCD) through incorporating iterative design and development, participatory action research by centering partnerships with end-users to bring about meaningful change with them rather than for them, and implementation science through a focus on understanding how best to implement and sustain a DMHI” (p. 2)			P23
	Participatory Design (PD):it involves end-users, researchers, and developers as equal partners throughout all stages of product development, from conception to completion^P5, P9^. It is based on iterative design cycles where stakeholders collaboratively produce knowledge and shape the final outcome, with end-users sharing equal responsibility for results – not as consultants, but as co-creators^P5^. PD aims to align usability with user experience and clinical best practices, producing interventions that are functional, engaging, and suited to real-life settings^P9^.			P5, P9

## Discussion

The findings of this scoping review reveal a marked increase in publications since 2022, suggesting a growing academic and policy interest in collaborative approaches to mental health research involving young people. This upward trend likely reflects several converging developments, including the increasing institutionalisation and policy prioritisation of PPI and RRI [27, 28, 30], growing recognition of the importance of involving young people in mental health research [13–15] and the rapid expansion of digital mental health research and by extension intervention development [7, 18, 39, 45–47]. The COVID-19 pandemic may also have contributed by intensifying concerns about young people’s mental health and accelerating the use of remote and digitally mediated approaches to research, youth support, and the active involvement of young people [60, 61]. Taken together, these developments may have increased both the opportunities and expectations for young people to contribute as collaborators who are actively involved in shaping mental health-related research and interventions. However, this observed increase must be interpreted with a degree of caution, given the conceptual ambiguity and inconsistent application of these approaches in the reviewed studies [8]. This concern has also been highlighted by Williams et al. [21] and Palmer et al. [22], who discuss the notions of “cobiquity” (i.e., the widespread and often indiscriminate use of “co-” language that conflates different forms of collaboration and blurs the distinctive values of co-production) and a broader “participatory zeitgeist (i.e., ‘trendiness’)” surrounding such approaches.

The included studies were retrieved from a broad range of multidisciplinary bibliographic databases, encompassing health sciences, psychology, education, and the social sciences. This diverse distribution of sources underscores the inherently cross-sectoral nature of youth involvement in mental health research and the need for integrative approaches to evaluate how PPI and RRI have been operationalised. Nonetheless, the results reveal a clear dominance of PPI over RRI in the literature, with nearly 70% of the included studies retrieved using the PPI search strategy. This trend indicates that PPI remains the more established and widely applied approach in mental health research, particularly in English-speaking contexts, which is consistent with earlier research [9, 62]. While RRI values such as reflexivity, inclusion, and responsiveness were often present, they were rarely defined or applied explicitly, which hinders conceptual clarity and the meaningful comparison of approaches.

Another critical finding concerns the geographical distribution of the literature. Most studies originated from high-income countries in the Global North, particularly English-speaking countries such as the United Kingdom, Australia, Canada, and the United States. This concentration may reflect comparatively stronger research infrastructure, dedicated funding streams, institutional support for PPI and related involvement practices, and publication systems in which such work is more routinely recognised and reported. It may also reflect the historical institutionalisation of PPI terminology in countries such as the United Kingdom, alongside the greater visibility of English-language research in the databases searched. However, this pattern raises concerns about the global applicability of current evidence and suggests a need to expand research efforts in underrepresented regions. It also reflects the wider problem of WEIRD evidence, whereby knowledge generated predominantly in Western, Educated, Industrialised, Rich, and Democratic settings may be treated as broadly applicable despite being shaped by particular institutional, cultural, and socio-economic conditions [63–65]. As a result, current models of youth involvement may insufficiently capture differences in mental health systems, family and community relationships, understandings of youth agency, as well as locally grounded forms of collaboration. Hence, it is, therefore, essential to acknowledge that the very lexicon of PPI and RRI is grounded in Global North epistemologies and institutional structures, which may not reflect how collaborative principles are understood or enacted in Global South contexts. Furthermore, the exclusive inclusion of English-language articles may have limited the identification of relevant studies from non-English-speaking regions, thereby reinforcing epistemic exclusion and potentially underestimating involvement practices expressed through different terminologies or publication traditions.

Importantly, the limited operationalisation of PPI and RRI across the reviewed studies is not merely a conceptual gap, it has concrete implications for equity and justice in research involving young people. Aligned with the reflections made by O’Mara-Eves et al. [23], Palmer et al. [22], Staley [66] and Williams et al. [21], without consistency and precision in defining the type and level of involvement, it becomes difficult to implement and evaluate youth involvement effectively. This vagueness can reproduce tokenistic practices and hinder the capacity of collaborative processes to address intersectional barriers experienced by young people, such as socio-economic disadvantage, gender inequality, racism, religious marginalisation, or geographic isolation [8, 11, 21, 22]. In this sense, operational clarity is not simply a methodological necessity but a condition for enabling transformative and inclusive practices [62, 67].

The feasibility and authenticity of large-scale youth involvement also emerged as a point of critical reflection. A considerable number of studies (i.e., P1, P2, P5, P8, P17, P18, P19, P23, P25, P27, P28) reported involving more than 28 young people, with some exceeding 80 young people and one even had over 300 youths. While youth involvement is essential, previous literature has highlighted the resource-demanding nature of meaningful involvement [4, 8, 9, 11, 20, 23, 68]. It requires substantial time, funding, training, and reflexive practices to support genuine collaboration. As Farr et al. [69] noted, ongoing reflection on group processes is critical but often constrained by institutional limitations. Given these demands, the feasibility of achieving deep, dialogic involvement with large numbers of young people remains questionable and risks superficial or diluted involvement if not adequately resourced and supported. Moreover, considering this review’s scope (youth from 10 to 25 years old), there is a wide developmental span, within which expectations, capacities, and support needs may vary significantly. Additionally, young people often engage in projects over extended periods, during which they undergo substantial cognitive, emotional, and social development. A young person who joins a project at age 10 and remains involved for three years, for example, might not only acquire research-related skills but will also transition into a markedly different life stage, requiring evolving forms of support, recognition, and engagement to ensure sustained and meaningful involvement [4, 8].

That said, the large numbers reported in some studies may reflect the involvement of different groups of young people at various stages for distinct purposes. For example, in the case of P28, a small group was involved in the co-design and co-production phases, while a larger group was involved during the evaluation stage. This illustrates the different levels of involvement discussed by O’Mara-Eves et al. [23] and SCIE [70], specifically: *transformative*, when young people share decision-making and reciprocal expertise that reoriented the aims of the research (aligned with what is recognised as ‘co-production’); *intermediate*, when they are actively involved and mutually recognised but with limited decision-sharing (e.g., helping to co-develop and champion research materials); and *descriptive*, when they inform or engage with the project without sharing decisions (e.g., providing consultation or feedback at specific stages of the project).

Another significant issue pertains to the recognition of young people’s contributions. The review identified one case in which young people were positioned as co-researchers but they were not explicitly listed as co-authors. However, it was not clear if the young people had been given an option to be credited or not. Either way, this inconsistency points to a broader ethical concern about the acknowledgement of substantive youth contributions. As Green and Johns [71] argue, involvement without a redistribution of power maintains existing hierarchies and fails to challenge dominant research paradigms. The literature supports the view that co-authorship should be considered when young people contribute meaningfully to the research design, data collection, analysis, or interpretation [9, 72]. Authorship is not merely symbolic; it reflects ethical commitments to equity, recognition, and the redistribution of intellectual credit. Drawing from our own experience, we argue that researchers should actively address power asymmetries and offer young people the opportunity to be listed as co-authors, where appropriate, ensuring that this choice is informed, voluntary, and supported throughout the process [8, 11]. We believe that authors have a responsibility to maintain transparency wherever possible in their publications, particularly regarding conversations held with young people about recognition, and to clearly document the preferences expressed.

Finally, the findings reinforce the centrality of power relations and intersectional dynamics in collaborative youth research. As noted by Fernandes et al. [44], youth-adult hierarchies often remain unaddressed, risking tokenism and undermining the potential of shared decision-making. Similarly, Papageorgiou et al. [62] has emphasised that power is not only hierarchical but also shaped by race, class, disability, and educational background. These dynamics can generate discomfort among both adult researchers and young people, sometimes misinterpreted as interpersonal conflict rather than systemic inequality. To mitigate these issues, it is vital to recognise that, for instance, co-production is not suitable for all settings or individuals without theoretical alignment or sufficient preparation [4, 72]. Researchers must build inclusive environments where young people can exercise agency and negotiate their roles meaningfully [11].

In sum, while the reviewed studies reflect encouraging trends in youth involvement, they also highlight significant conceptual, ethical, and practical gaps in the field. Strengthening operational clarity, promoting epistemic diversity, and addressing power asymmetries are essential for advancing truly responsible and collaborative mental health research with young people.

### Implications for reporting and evaluating youth involvement

The results of this review point to several practice implications for how youth involvement is designed, reported, and evaluated in mental health research. These implications can be understood across three interrelated domains: (1) conceptual and definitional clarity, (2) developmental and intersectional nuance in ‘meaningful’ involvement, and (3) specifying levels of involvement and strengthening reporting standards:

1. **Conceptual and definitional clarity**: the limited definitional clarity and sparse reporting on how involvement approaches were actually applied in the included studies have significant implications for both implementation and evaluation. Meaningful involvement in youth mental health research is inherently high-maintenance and resource-demanding, requiring time, funding, training, and sustained relational work. When projects do not clearly define whether they are using, for example, PPI, RRI or related practices such as co-design or participatory research, and do not describe how these were implemented in practice (or the epistemological grounds and assumptions on which they are based), it becomes difficult to evidence the specific outcomes of involvement. This, in turn, makes it harder to convincingly communicate the added value and transformative potential of youth involvement to organisations (e.g., universities, services, hospitals) and funders, who often seek demonstrable returns on the additional resources invested in collaborative approaches;
2. **Developmental and intersectional nuance in ‘meaningful’ involvement**: it is also critical to acknowledge the complex and uncertain nature of what counts as “meaningful” involvement in youth mental health. Within the broad 10–25-year age range considered in this review, young people’s developmental stages, capacities, expectations, and support needs vary substantially. For instance, meaningful involvement for a 10-year-old may centre on feeling safe, heard, and appropriately supported in discrete activities, whereas for a young adult it may involve sustained co-leadership, authorship, and strategic influence over a project. These dimensions are further shaped by intersecting factors such as race, gender identity, disability, socio-economic position, and migration or linguistic status. Clear reporting on who was involved, in what ways, and under which conditions is therefore essential not only for transparency, but also for recognising that ‘good’ or ‘meaningful’ involvement may legitimately look different across age groups and intersectional positions;
3. **Levels of involvement and reporting standards**: in youth mental health research, differentiating levels of involvement is particularly useful for both implementation and evaluation. Building on the distinctions proposed by O’Mara-Eves et al. [23] and SCIE [70], this review illustrates the value of distinguishing between transformative, intermediate, and descriptive levels of involvement. We do not argue that all projects must achieve a transformative level. Rather, we suggest that future studies should explicitly state the intended level of involvement (that is, transformative, intermediate, or descriptive), state the concrete practices used to enact it, and reflect on the extent to which these intentions were realised in practice. Implicit in this recommendation is the view that journal reporting structures, including section templates and word counts, also have a role to play in supporting, prompting, and, where appropriate, requiring such clarity. Even modest adjustments (for example, routinely asking authors for a brief statement on the intended level of involvement and the practices used to realise it) could help ensure that information about youth involvement is not ‘crowded out’ by a paper’s space or word constraints. Such clarity would enable more nuanced evaluation of outcomes, allow fairer comparison across projects, and support the development of context-sensitive, realistic expectations for youth involvement in mental health research.

### Strengths, limitations and recommendations for future studies

The inclusion of diverse approaches and definitions for PPI and RRI enabled a useful analysis of conceptual clarity, methodological practices and ethical dimensions relating to youth involvement in youth mental health. The broad eligibility criteria applied to mental health-related projects also enabled the review to capture youth involvement across varied project aims, disciplinary contexts, methodologies and settings, in keeping with the exploratory and mapping purposes of a scoping review. However, this breadth may have contributed to the heterogeneity of the included literature and limited direct comparability across studies. Nonetheless, limitations must be acknowledged. First, the review was restricted to English-language publications, which may have excluded relevant studies from non-English-speaking countries, particularly those in the Global South. Second, despite the broad search strategy and the inclusion of terms relating to PPI, RRI, co-production, participatory action research and co-design, the inconsistent and overlapping terminology used by authors to describe youth involvement may have resulted in relevant studies being missed. This risk may be particularly pertinent where collaborative practices were described using locally specific, emerging or inadequately indexed terminology. Third, although the review provides a valuable overview of reported practices, it is constrained by the level of detail available in the publications, which was often limited or inconsistently reported, particularly regarding young people’s demographic characteristics and the nature of their involvement. Consequently, the findings reflect only what has been documented in the published literature, rather than the full breadth of processes and practices in the ‘real world’ of the youth mental health field. Given the scope, objectives, intended purpose and resource limitations of a single review, it was not possible to examine in depth all developmental, intersectional and structural factors that may shape young people’s involvement, which constitutes a limitation of the review. Future reviews could examine whether particular features of qualitative designs may facilitate or make youth involvement more visible, while avoiding assumptions that the predominance of qualitative studies necessarily reflects a greater suitability for PPI or RRI. Subsequent reviews could also compare how the reported benefits and challenges of involvement vary according to: study aims; research questions; and methodological designs and project types. This could include exploring whether intervention-development studies differ from research, service-evaluation or product- and technology-development initiatives. Such analyses should situate their findings within the wider literature on the practical, methodological and ethical challenges of Patient and Public Involvement and Engagement (PPIE) in youth research. Nevertheless, given the importance of these issues, future studies could examine more explicitly how developmental differences across the broad 10–25-year age range, as well as changes in young people’s capacities, expectations and support needs over the course of a project, can influence the implementation of PPI and RRI approaches. This is important in order to avoid treating young people as a homogeneous population and to consider how developmental factors intersect with social, cultural and structural characteristics, which in turn shape what constitutes appropriate and meaningful involvement, including in terms of the potential barriers and challenges related to meaningfully involving young people under 16 years of age where parental/guardian consent is routinely required. Particular attention should be given to the inclusion and experiences of marginalised, minoritised or underrepresented groups, including young people from ethnic minority communities, those experiencing socio-economic disadvantage, young people with physical disabilities and those with lived experience of mental illness – it is important to examine not only whether these groups are represented, but also how intersecting inequalities, accessibility requirements, stigma, power relations and institutional practices can influence their opportunities to contribute, inform decision-making and sustain meaningful involvement. Future research could also examine whether and how established youth involvement frameworks (e.g., Hart’s Ladder of Participation and Lundy’s Model of Participation) could potentially complement PPI- and RRI-oriented youth involvement.

## Conclusions

This scoping review maps how PPI and RRI approaches have been conceptualised and operationalised in mental health-related projects involving young people. The findings indicate that while youth involvement is gaining traction in research, especially through PPI practices in high-income English-speaking countries, the evidence remains geographically concentrated in a small number of countries and marked by conceptual ambiguity, inconsistent terminology and uneven reporting.

The review reveals that PPI is more frequently cited and applied than RRI. Across the literature, PPI, RRI and related collaborative terms were frequently insufficiently defined or conceptually blended, making it difficult to determine how the reported approaches were operationalised and how much influence young people exercised. Importantly, the label assigned to an approach should not be treated as evidence of the depth, quality or distribution of decision-making power achieved in practice. Moreover, the dominance of Global North contexts, coupled with the English-language focus and the inclusion criterion of primary data studies only, limits the transferability of current knowledge and may have obscured conceptual, theoretical and locally grounded contributions from non-English-speaking and underrepresented regions.

Future studies should therefore adopt more inclusive linguistic, geographical and epistemological lenses and examine how youth involvement is understood and enacted across underrepresented regions, particularly the Global South. Researchers should also clearly distinguish the overarching approach adopted, the specific activities and roles undertaken by young people, and the level of influence and decision-making authority achieved.

The review also highlights the resource-intensive and relational nature of meaningful youth collaboration, raising questions about the feasibility and authenticity of large-scale youth involvement, the risks of tokenism and the consistent recognition of substantive youth contributions – for instance, authors could state in the authorship summary that young people were offered authorship but declined or preferred to only be recognised in the acknowledgements (by name or in a de-identified manner). Addressing these challenges requires adequate resources and support, transparent reporting and authorship practices, attention to power asymmetries, and inclusive and reflexive research environments in which young people can meaningfully shape both processes and outputs.

Overall, advancing responsible and collaborative youth involvement in mental health research requires more than the adoption of collaborative-sounding labels. It requires explicit conceptual and epistemological foundations, transparent operationalisation and reporting, recognition of developmental and intersectional differences, and genuine opportunities for young people to exercise influence and receive appropriate recognition for their contributions.

## Supplementary Information

Below is the link to the electronic supplementary material.

Supplementary Material 1

Supplementary Material 2

Supplementary Material 3

Supplementary Material 4

Supplementary Material 5

## Data Availability

All data generated or analysed during this study are included in this published article and its supplementary information files.

## Footnotes

1 Drawing on our previous work on PPI/RRI in youth mental health and on coproduction with young people [8,11,39], we understand “meaningful involvement” as forms of youth involvement that go beyond one-off consultation. Such involvement is characterised by early and, ideally, sustained involvement across project stages, clear opportunities to influence agendas and decisions, reciprocal recognition of lived and experiential expertise, appropriate renumeration, support and safeguarding (including attention to emotional labour and accessibility), and a genuine capacity for young people’s contributions to shape both processes and outputs, rather than being merely tokenistic.

2 In this review, a “mental health-related project” refers to any empirical research, intervention-development, service-evaluation, policy-development, or product/technology-development initiative in which mental health or mental well-being was a primary focus. This includes projects across mental health: promotion; prevention; assessment; treatment or support; service delivery; and mental health digital innovation across clinical, community, educational and online settings, while excluding projects in which mental health was only incidental.

3 Within Digital Youth, the ‘Youth Co-chair’ is a formal role for young people who share leadership with researchers. They help set agendas, facilitate meetings, represent youth perspectives, and ensure that discussions and decisions reflect intergenerational equity. By doing so, Youth Co-chairs move beyond tokenism, redistribute power, and position young people as genuine partners and leaders in youth mental health and digital innovation.

## Abbreviations

AREA: Anticipate, reflect, engage, act
ABM: Arts-based methods
CADIMA: Central access database for the impact assessment of genetic improvement technologies
JBI: Joanna briggs institute
NIHR: National institute for health and care research
PCCS: Participants, concept, context, and sources
PPI: Patient and public involvement
PRISMA-ScR: Preferred reporting items for systematic reviews and meta-analyses extension for scoping reviews
RRI: Responsible research and innovation
SSI: Single-session intervention
UK: United kingdom
YP: Young people
YPAG: Young people advisory group
