# Supplementary material for: Conceptualising and operationalising ‘patient and public involvement’ (PPI) and ‘responsible research and innovation’ (RRI) in youth mental health research: a scoping review
Source: Res Involv Engagem. 2026 Sep 24;12:156. doi: 10.1186/s40900-026-00969-3 (PMC13602541; doi:10.1186/s40900-026-00969-3)
Supplement: Supplementary file 1 [file 40900_2026_969_MOESM1_ESM.docx]

# Additional File 1: Search Strategy

**Article:** Involving Young People in Mental Health Research: A Scoping Review of ‘Patient and Public Involvement’ (PP)I and ‘Responsible Research and Innovation’ (RRI) Approaches

**Journal:** Research Involvement and Engagement

**Authors:** Josimar Antônio de Alcântara Mendes, Mathijs Lucassen, Alex Adams, Ayan Mahamud, Lucy Hitcham, Sachiyo Ito-Jaeger, Christine Aicardi, Emma Nielsen, Lily Roberts, Ellen Townsend and Marina Jirotka

|  | **POPULATION** | **CONCEPT/CONTENT** | **CONTEXT** |
| --- | --- | --- | --- |
| **PPI** | “young people” OR adolescen* OR youth OR teenage* OR teens | “patient and public involvement” OR “patient involve*” OR “patient engage*” OR “user involve*” OR “user engage*” OR “participant engage*” OR “participant involve*” OR  “public engage*” OR “public involve*” OR “expert by experience” OR “co-design*” OR codesign* OR “co-produc*” OR coproduc* OR co-creat* OR cocreat* | “mental health” OR  “mental wel*” OR “emotional health” OR “emotional wel*” OR “psychological health” OR “psychological wel*”  OR “mental health service”  OR “mental health care” OR “psycholog*” OR “psychiat*” |
| **RRI** | “young people” OR adolescen* OR youth OR teenage* OR teens | “responsible research and innovation” OR “responsible innovat*” OR “responsible engagement” OR “responsible involvement” OR “responsible research” OR “participat* research” OR “peer research” OR “citizen science” OR “citizen participation” OR human-cent* OR “participat* design” OR “technology assess*” OR “ethical, legal and social aspects” OR “ethical, legal, and social implications” OR “user-led” OR “user-cent*” OR “client-led” or “patient-led” OR “client-cent*” OR “patient-cent*” | “mental health” OR  “mental wel*” “emotional health” OR “emotional wel*” OR “psychological health” OR “psychological wel*”  OR “mental health service”  OR “mental health care” OR “psycholog*” OR “psychiat*” |
