# Supplementary material for: Conceptualising and operationalising ‘patient and public involvement’ (PPI) and ‘responsible research and innovation’ (RRI) in youth mental health research: a scoping review
Source: Res Involv Engagem. 2026 Sep 24;12:156. doi: 10.1186/s40900-026-00969-3 (PMC13602541; doi:10.1186/s40900-026-00969-3)
Supplement: Supplementary file 2 [file 40900_2026_969_MOESM2_ESM.docx]

# **Additional File 2: Excluded Full-text Articles**

**Article:** Involving Young People in Mental Health Research: A Scoping Review of ‘Patient and Public Involvement’ (PP)I and ‘Responsible Research and Innovation’ (RRI) Approaches

**Journal:** Research Involvement and Engagement

**Authors:** Josimar Antônio de Alcântara Mendes, Mathijs Lucassen, Alex Adams, Ayan Mahamud, Lucy Hitcham, Sachiyo Ito-Jaeger, Christine Aicardi, Emma Nielsen, Lily Roberts, Ellen Townsend and Marina Jirotka

| **Paper's ID:** | **Paper's title:** | **Authors:** | **Year:** | **Place:** | **Does it present, describe or evaluate, via primary data, mental health-related projects (e.g., research, intervention, product development) collaborating with young people (10 to 25 years)?** | **Justification** |
| --- | --- | --- | --- | --- | --- | --- |
| **4** | Evaluating Web-Based Care for Mental Health and Substance Use Issues for Lesbian, Gay, Bisexual, Transgender, Queer, Questioning, and 2-Spirit Y ouths in the Context of the COVID-19 Pandemic: Community-Based Participatory Research Study | Michael Chaiton; Rachel Thorburn; Emily Chan; Ilana Copeland; Chieng Luphuyong; Patrick Feng | 2023 | Canada | No | They said “Every step of the research process, from conception to analysis, included LGBTQ2S+ youths on the research and facilitation team”. However, they only provide information about YP involvement as research participants. |
| **6** | Using cocreated visually informed community mental health education in low‐ and middle‐income countries: A case study of youth substance misuse in Assam, India | Raginie Duara, Diptarup Chowdhury, Ratul Dey, Sangeeta Goswami, Anna Madill | 2022 | UK | No | They mention ‘cocreation’, but YP were only research participants. |
| **7** | A Coproduced Traumatic Bereavement Therapeutic Group for Moroccan Adolescents Affected by the Grenfell Tower Fire | Amell El Guenuni, Rajaa Chellaty, Becky Cantonz, Fatima El Guenunix and Jeyda Hammad | 2022 | UK | No | They refer to cocreation, but the paper just describes a “culturally adapted and sensitive” intervention. YP did not take part in the process of planning, designing, and/or delivering the therapeutic group. |
| **8** | Finding harmony within dissonance: Engaging patients, family/ caregivers and service providers in research to fundamentally restructure relationships through integrative dynamics | Gillian Mulvale, Jenn Green, Ashleigh Miatello, Ann E. Cassidy, Terry Martens | 2019 | Canada | No | YP took part only as research participants. |
| **9** | ‘It reshaped how I will do research’: A qualitative exploration of team members’ experiences with youth and family engagement in a randomized controlled trial | Natasha Y. Sheikhan, Lisa D. Hawke, Kristin Cleverley, Karleigh Darnay, Peter Szatmari | 2020 | Canada | No | YP took part only as research participants. |
| **10** | ‘We’re all in the same boat’: An Interpretative Phenomenological Analysis study of experiences of being an ‘expert’ during patient and public involvement within Child and Adolescent Mental Health Services (CAMHS) | Samiran Idrees, Samantha Hartley, Jasmine Heath Hearn | 2020 | UK | No | YP took part only as research participants. |
| **11** | Making sense of it together: Youth & families co-create sensory modulation assessment and intervention in community mental health settings to optimise daily life | Phoebe Williamson, Priscilla Ennals | 2020 | Australia | No | YP took part only as research participants. |
| **12** | Young parents’ experiences of a multi-agency young families project: Findings from a co-produced study | Susanne Darra, Michael R.M. Ward, Catherine Jones, Sara Jones | 2020 | UK | No | YP took part only as research participants. |
| **13** | Co-designing the InnoWell Platform to deliver the right mental health care first time to regional youth | Shelley C. Rowe, Tracey A. Davenport, Tanya A. Jackson, Jennifer Melsness, Jenny Sinclair, Michael A. Easton, Antonia Ottavio, Ian B. Hickie | 2020 | Australia | No | YP took part only as research participants. |
| **17** | ‘…it shows that there’s no limits’: the psychological impact of co-production for experts by experience working in youth mental health | Claire Mayer, Karen McKenzie | 2017 | UK | No | YP took part only as research participants. |
| **18** | Telling stories: learning from patients’ and families’ experiences of specialist child and adolescent mental health servicesijcs_904 | Alka S. Ahuja, Richard Williams | 2010 | UK | No | YP took part only as research participants. |
| **19** | It Takes a Village to Deliver and Test Child and Family-Focused Services | Mary M. McKay, Geetha Gopalan, Lydia M. Franco, Kosta Kalogerogiannis, Mari Umpierre, Orly Olshtain-Mann, William Bannon, Laura Elwyn, Leah Goldstein | 2010 | USA | No | Just describe MH services - no involvement of YP |
| **22** | Enhancing knowledge of mental health issues among children’s nursing students: evaluation of a service user-led workshop | Mary Patricia Brady, Jayne Price, Ann Ooms et al. | 2022 | UK | No | They mention that young people were involved, but they do not provide further information regarding sociodemographic info or the extent of their participation. |
| **23** | Exploring young people’s perceptions of health and inequality through art: a co-produced qualitative study | Laura Tinner | 2022 | UK | No | Conference abstract |
| **24** | Indigenous youth and service provider perceptions of the availability and appropriateness of services to promote Indigenous youth mental health | Janya McCalman, Tina McDonald, Semara Jose, Ruth Fagan, Kelly Walker, Rodney Collins | 2021 | Australia | No | Conference abstract |
| **25** | Recommendations for Designing Health Information Technologies for Mental Health Drawn From Self-Determination Theory and Co-design With Culturally Diverse Populations: Template Analysis | Vanessa Wan Sze Cheng, Sarah E. Piper, Antonia Ottavio, Tracey A. Davenport, Ian B. Hickie | 2021 | Australia | No | They mention that young people were involved, but they do not provide further information regarding sociodemographic (number of YP, age, gender, background) or the extent of their participation. |
| **27** | Youth Perspectives on Barriers and Opportunities for the Development of a Peer Support Model to Promote Mental Health and Prevent Suicide | Jackie Libon, Jarlo Alganion, Carla Hilario | 2023 | Canada | No | They mention co-design but do not offer further details, and YP were primarily research participants. |
| **29** | A COMPASS for Navigating Relationships in Co-Production Processes Involving Vulnerable Populations | Gillian Mulvale, Ashleigh Miatello, Jenn Green, Maxwell Tran, Christina Roussakis & Alison Mulvale | 2021 | Canada | No | Study run with researchers |
| **30** | Co-designing Services for Youth With Mental Health Issues: Novel Elicitation Approaches | Gillian Mulvale, Sandra Moll, Ashleigh Miatello, Louise Murray-Leung, Karlie Rogerson, Roberto B. Sassi | 2019 | Canada | No | YP took part only as research participants. |
| **32** | User-Centered Design of a Gamified Mental Health App for Adolescents in Sub-Saharan Africa: Multicycle Usability Testing Study | Julia R. Pozuelo, Bianca D. Moffett, Meghan Davis, Alan Stein, Halley Cohen, Michelle G. Craske, Meriam Maritze, Princess Makhubela, Christine Nabulumba, Doreen Sikoti, Kathleen Kahn, Tholene Sodi, DoBAt & Ebikolwa Consortium, Alastair van Heerden, Heather A. O’Mahen | 2023 | South Africa & Uganda | No | Information about the age range is not accurate - the information is incomplete or confusing |
| **34** | Determining Priorities in the Aboriginal and Islander Mental Health Initiative for Y outh App Second Phase Participatory Design Project: Qualitative Study and Narrative Literature Review | Josie Povey, Michelle Sweet, Tricia Nagel, Anne Lowell, Fiona Shand, Jahdai Vigona, Kylie M. Dingwall | 2022 | Australia | No | Information about the extensiveness of youth participation is not clear |
| **35** | Improving mental health literacy among young people aged 11–15 years in Java, Indonesia: the co‑development of a culturally‑appropriate, user‑centred resource (The IMPeTUs Intervention) | Helen Brooks, Armaji Kamaludi Syarif, Rebecca Pedley, Irman Irmansyah, Benny Prawira, Karina Lovell, Cicih Opitasari, Adam Ardisasmita, Ira Savitri Tanjung, Laoise Renwick, Soraya Salim, Penny Bee | 2021 | Indonesia | No | Does not state the age range of YP involved and focus on reporting the literature review process |
| **36** | Development of the Niggle App for Supporting Y oung People on Their Dynamic Journey to Well-being: Co-design and Qualitative Research Study | Stoyan R. Stoyanov, Oksana Zelenko, Aleksandra Staneva, David J. Kavanagh, Calvin Smith, Gavin Sade, Jessica Cheers, Leanne Hides | 2021 | Australia | No | Even though they call it “participatory design workshops”, YP are involved mainly as research participants |
| **37** | ‘Getting our voices out there’: acceptability of a mental health participation programme for young people with out of home care experience in Australia | Jessie Rafeld, Kristen Moeller-Saxone, Sue Cotton, Simon Rice, Katherine Monson, Carol Harvey, Helen Herrman | 2020 | Australia | No | YP involved just as research participants |
| **38** | Informing the development of an E-platform for monitoring wellbeing in schools: involving young people in a co-design process | Claire Grant, Emily Widnall, Lauren Cross, Emily Simonoff, Johnny Downs | 2020 | UK | No | Even though they call it ‘YPAG sessions’, YP are involved mainly as research participants |
| **39** | Opening the door: inviting youth and parent perspectives on youth mental health emergency department use | Leslie Anne Campbell, David Lovas, Ellen Withers, Kylie Peacock | 2020 | Canada | No | Even though they mention YPAG, YP were research participants. |
| **40** | What makes for effective, sustainable youth engagement in knowledge mobilization? A perspective for health services | Eugenia Canas, Lisa Lachance, David Phipps, Cherrilyn Che Birchwood | 2019 | Canada | No | Even though they mention YPAG, YP were involved just as research participants. |
| **40** | The Importance of User Segmentation for Designing Digital Therapy for Adolescent Mental Health: Findings From Scoping Processes | Theresa Fleming, Sally Merry, Karolina Stasiak, Sarah Hopkins, Tony Patolo, Stacey Ruru, Manusiu Latu, Matthew Shepherd, Grant Christie, Felicity Goodyear-Smith | 2019 | New Zealand | No | YP took part only as research participants. |
| **42** | Young adults’ perceptions of using wearables, social media and other technologies to detect worsening mental health: A qualitative study | Lindsay H. Dewa, Mary Lavelle, Katy Pickles, Caroline Kalorkoti, Jack Jaques, Sofia Pappa, Paul Aylin | 2019 | UK | No | No information about the age range (or demographics) of YP that they called ‘co-researchers’ |
| **43** | Design and Development of the “PODAdventures” Smartphone Game: A Blended Problem-Solving Intervention for Adolescent Mental Health in India | Pattie P. Gonsalves, Eleanor S. Hodgson, Avinash Kumar, Tiara Aurora, Yash Chandak, Rhea Sharma, Daniel Michelson, Vikram Patel | 2019 | India | No | YP took part only as research participants. |
| **44** | Prioritizing young people’s emotional health support needs via participatory research | S. E. Kendal, L. Milnes, H. Welsby, S. Pryjmachuk | 2017 | UK | No | Even though they mentioned ‘participatory research’, there is not much information regarding the extensiveness of YP participation beyond ‘research participation’. |
| **45** | Development, preliminary validation and reliability of the colourful ‘My Feelings Form’ self-report for young children | Aisling Mulligan, Natthaphol Sresthaporn, Sinead Mulroy, Tara Rudd, Anne Coffey, Maria Joyce, Veselina Gadancheva, Caoimhe Glancy | 2023 | Ireland | No | Focus on young children - below 12 years old - and research participation |
| **46** | “A peer support worker can really be there supporting the youth throughout the whole process”: a qualitative study exploring the role of peer support in providing substance use services to youth | Roxanne Turuba, Ciara Toddington, Miranda Tymoschuk, Anurada Amarasekera, Amanda Madeleine Howard, Violet Brockmann, Corinne Tallon, Sarah Irving, Steve Mathias, J. L. Henderson, Skye Barbic | 2023 | Canada | No | Even though they refer to ‘Participatory action research methods’, the YP involvement was more related to research participation. |
| **47** | The League: A person‐centred approach to the development of social robotics for paediatric anxiety | Jill A. Dosso, Jaya N. Kailley, Julie M. Robillard | 2023 | Canada | No | Information about the involvement of young people and their characteristics is limited. |
| **49** | Development of a school-based programme for mental health promotion and prevention among adolescents in Nepal and South Africa | Christina A. Laurenzi, Stefani du Toit, Tatenda Mawoyo, Nagendra P. Luitel, Mark J. D. Jordans, Indira Pradhan, Claire van der Westhuizen, G.J. Melendez-Torres, Jemma Hawkins, Graham Moore, Rhiannon Evans, Crick Lund, David A. Ross, Joanna Lai, Chiara Servili, Mark Tomlinson, Sarah Skeen | 2024 | Nepal and South Africa | No | YP took part only as research participants. |
| **51** | Juveniles Accessing Mental health Services: A Novel Approach to Enhance Mental Health Among Vulnerable Adolescents | Shiva Prakash Srinivasan, Aloka Datta Behra, Chiranjeevi Arumugam, Protush Panda, Rangeela E., Smriti Smitha Nayak, Padmavati Ramachandran | 2023 | India | No | YP took part only as research participants. |
| **52** | Perceptions of the key components of effective, acceptable and accessible services for children and young people experiencing common mental health problems: a qualitative study | Susan Kirk, Claire Fraser, Nicola Evans, Rhiannon Lane, Jodie Crooks, Georgia Naughton, Steven Pryjmachuk | 2023 | UK | No | Regarding PPI, they just refer to the involvement of “a group of six young adults with lived experience of mental health issues” without stating their age range or other sociodemographic factors. Apart from this, young people were involved just as research participants. |
| **53** | A community‑based approach to identifying and prioritising young people’s mental health needs in their local communities | Ediane Santana de Lima, Cristina Preece, Katie Potter, Ellen Goddard, Julian Edbrooke-Childs, Tim Hobbs, Peter Fonagy | 2023 | UK | No | Even though they mention that they have used a “community-based participatory approach”, YP were involved as research participants. |
| **54** | “Don’t tell me how to tell my story”: Exploring young people’s perceptions around what it means to ‘feel (mis)understood’ by adults in supporting roles | Emily Cunningham, Isla Jamieson-MacKenzie, Christina McMellon, Martha McCallin, Myada Eltiraifi, Levi Smith, Katie Hepburn | 2024 | UK | No | They touch on the topic of ‘mental health’, but it is not the paper’s focus. |
| **55** | A qualitative evaluation of a co‐design process involving young people at risk of suicide | Michelle Kehoe, Rick Whitehead, Kathleen de Boer, Denny Meyer, Liza Hopkins, Maja Nedeljkovic | 2024 | Australia | No | No information regarding YP’s age range or their sociodemographics is presented; only one young person was involved. |
| **57** | The potential of virtual reality to support adolescent mental well-being in schools: A UK co-design and proof-of-concept study | Siobhan Hugh-Jones, Michelle Ulor, Trish Nugent, Sophie Walshe, Mike Kirk | 2023 | UK | No | YP took part only as research participants. |
| **58** | “I literally had no support”: barriers and facilitators to supporting the psychosocial wellbeing of young people with mental illness in Tasmania, Australia | Melissa Savaglio, Marie B. H. Yap, Toni Smith, Ash Vincent, Helen Skouteris | 2023 | Australia | No | YP took part only as research participants. |
| **59** | An Artificial Therapist (Manage Y our Life Online) to Support the Mental Health of Y outh: Co-Design and Case Series | Aimee-Rose Wrightson-Hester, Georgia Anderson, Joel Dunstan, Peter M. McEvoy, Christopher J. Sutton, Bronwyn Myers, Sarah Egan, Sara Tai, Melanie Johnston-Hollitt, Wai Chen, Tom Gedeon, Warren Mansell | 2023 | Australia | No | YP took part only as research participants. |
| **61** | A myth-busting mental health tour of the National Gallery in London: Facilitators and challenges to its development and evaluation | Simon Riches, Ruxandra Vasile, Natalie Steer, Anna Murray, Amber Goneni, Aleksandra Orehova, Rachel Temple, Rose Thompson, Fiona Houston & Helen L. Fisher | 2023 | UK | No | They generically refer to ‘young people’ without specifying their age range or other sociodemographic characteristics. |
| **64** | Youth experiences of co-designing a well- being intervention: reflections, learnings and recommendations | Dianne Vella-Brodrick, Kent Patrick, Rowan Jacques-Hamilton, Amanda Ng, Tan-Chyuan Chin, Meredith O’Connor, Nikki Rickard, Donna Cross & John Hattie | 2023 | Australia | No | YP are involved just as research participants - data collection focused on YP experience as ‘Youth Advisors’. |
| **66** | Early evaluation of the Children and Young People’s Mental Health Trailblazer programme: a rapid mixed-methods study | Jo Ellins, Lucy Hocking, Mustafa Al-Haboubi, Jenny Newbould, Sarah-Jane Fenton, Kelly Daniel, Stephanie Stockwell, Brandi Leach, Manbinder Sidhu, Jenny Bousfield, Gemma McKenna, Katie Saunders, Stephen O’Neill and Nicholas Mays | 2023 | UK | No | Even though it is published in a journal, it’s a research report, not a paper. |
| **67** | Development and Evaluation: A Behavioral Activation Mobile Application for Self-Management of Stress for College Students | Hyunjoo Na, Minjeong Jo, Chaerin Lee, Doyoon Kim | 2022 | South Korea | No | They just mentioned that “five nursing college students” helped to co-designed materials, but no further information regarding age range or demographics was provided; the other young people participated just as research participants. |
| **68** | Satisfaction, Perceived Usefulness, and Therapeutic Alliance as Correlates of Participant Engagement in a Pediatric Digital Mental Health Intervention: Cross-Sectional Questionnaire Study | Landry Huffman, Monika Roots, Kurt Roots, Darian Lawrence-Sidebottom, Jennifer Huberty, Clare Beatty, Amit Parikh, Rachael Guerra | 2023 | USA | No | YP took part only as research participants. |
| **69** | Heart Rate Variability Biofeedback to Treat Anxiety in Y oung People With Autism Spectrum Disorder: Findings From a Home-Based Pilot Study | Helen Coulter, Mark Donnelly, John Mallett, W. George Kernohan | 2022 | UK | No | Adults took part in the co-design process, and YP were involved only as research participants. |
| **70** | “What makes discovery college different?” a co- produced analysis of student experiences of discovery college | Richard Whitehead, Claire Harcla, Liza Hopkins & Ella Robinson-Clarke | 2024 | Australia | No | Even though they refer to ‘co-production’, YP were involved just as research participants. |
| **71** | “It cannot be boring!”: Developing a measure of function for young adults accessing integrated youth services | Skye Barbic, Emily Brooks, Natalia Lassak, Mo Khaleghi, Marco Zenone, Nikki Ow, Adelena Leon, Steve Mathias | 2022 | Canada | No | They refer to ‘youth research lead’ and ‘youth research assistant’, but no information about their age was provided; the other YP were involved as research participants. |
| **72** | The Availability, Appropriateness, and Integration of Services to Promote Indigenous Australian Youth Wellbeing and Mental Health: Indigenous Youth and Service Provider Perspectives | Janya Robyn McCalman, Ruth Fagan, Tina McDonald, Semara Jose, Paul Neal, Ilse Blignault, Deborah Askew, Yvonne Cadet-James | 2023 | Australia | No | The study was co-produced by a health service and not by YP. |
| **73** | Evidencing the Impact of Web-Based Coproduction With Y outh on Mental Health Research: Qualitative Findings From the MindKind Study | Blossom Fernandes, Lakshmi Neelakantan, Himani Shah, Sushmita Sumant, Pamela Y. Collins, Jennifer Velloza, Emily Bampton, Swetha Ranganathan, Refiloe Sibisi, Toiba Bashir, Joshua Bowes, Esther Larisa David, Harsimar Kaur, Umairah Malik, Issy Shannon, Suvlaxmi Gurumayum, Anne-Marie Burn, Solveig K. Sieberts, The MindKind Consortium, Mina Fazel | 2023 | India, South Africa, and the United Kingdom | No | They do not specify the number of YPs involved. They just mentioned ‘young people’ generically. |
| **74** | Adolescents accept digital mental health support in schools: A co-design and feasibility study of a school-based app for UK adolescents | Siobhan Hugh-Jones, Kirsty Pert, Sarah Kendal, Simon Eltringham, Chris Skelton, Nahel Yaziji, Robert West | 2022 | UK | No | They do not specify the number or the age range of YP involved in the co-designing process. They just inform the age range of YP involved in the feasibility test. |
| **76** | Experts by Experience: Qualitative Evaluation of Adolescent Participation in the Development of a Technological Intervention to Prevent Youth Suicide in Chile | Sara Hamilton Schilling, Alejandra Carreño, Eric Tapia, Franco Mascayano, Romina Pitronello, Felipe Santander, María José Jorquera, María Soledad Burrone, Ruben Vladimir Alvarado | 2021 | Chile | No | Even though they call YP ‘Group of Experts’, YP were involved just as research participants. |
| **78** | Co-producing to understand what matters to young people living in youth residential rehabilitation services | Priscilla Ennals, Kate Lessing, Rebecca Spies, Rebecca Egan, Philippa Hemus, Kathryn Droppert, Michael Tidhar, Tom Wood, Carolien van Dijk, Rachael Bride, Alison Asche, Sarah Bendall, Magenta Simmons | 2021 | Australia | No | Young people collaborating with the project were generically referred to as ‘young people’ - no other information was provided. The rest of the YP were involved as research participants. |
| **79** | A Cognitive Behavioral Therapy–, Biofeedback-, and Game-Based eHealth Intervention to Treat Anxiety in Children and Y oung People With Long-Term Physical Conditions (Starship Rescue): Co-design and Open Trial | Hiran Thabrew, Karolina Stasiak, Harshali Kumar, Tarique Naseem, Christopher Frampton, Sally Merry | 2021 | New Zealand | No | YP took part only as research participants. |
| **81** | Developing a Web-Based App to Assess Mental Health Difficulties in Secondary School Pupils: Qualitative User-Centered Design Study | Anne-Marie Burn, Tamsin J. Ford, Jan Stochl, Peter B. Jones, Joanna K. Anderson | 2022 | UK | No | YP took part only as research participants. |
| **82** | Co-creation and regional adaptation of a resilience-based universal whole-school program in five European regions | Roxanna Morote, Carlota Las Hayas, Irantzu Izco-Basurko, Frederick Anyan, Ane Fullaondo, Valeria Donisi, Antoni Zwiefka, Dora Gudrun Gudmundsdottir, Mette Marie Ledertoug, Anna S. Olafsdottir, Silvia Gabrielli, Sara Carbone, Iwona Mazur, Anna Królicka-Deręgowska, Hans Henrik Knoop, Nina Tange, Ingibjörg V. Kaldalóns, Bryndís Jóna Jónsdóttir, Ana González Pinto, Odin Hjemdal | 2022 | Spain, Italy, Poland, Denmark and Iceland | No | Even though they mention “participatory sessions”, YP were involved mainly as research participants. |
| **83** | Informing the Future of Integrated Digital and Clinical Mental Health Care: Synthesis of the Outcomes From Project Synergy | Haley M. LaMonica, Frank Iorfino, Grace Yeeun Lee, Sarah Piper, Jo-An Occhipinti, Tracey A. Davenport, Shane Cross, Alyssa Milton, Laura Ospina-Pinillos, Lisa Whittle, Shelley C. Rowe, Mitchell Dowling, Elizabeth Stewart, Antonia Ottavio, Samuel Hockey, Vanessa Wan Sze Cheng, Jane Burns, Elizabeth M. Scott, Ian B. Hickie | 2022 | Australia | No | YP took part only as research participants. |
| **85** | Co-­ designing for quality: Creating a user-­ driven tool to improve quality in youth mental health services | Christina L. Hackett, Gillian Mulvale, Ashleigh Miatello | 2018 | Canada | No | YP took part only as research participants. |
| **86** | An intervention to improve the quality of life in children of parents with serious mental illness: the Young SMILES feasibility RCT | Kathryn M Abel, Penny Bee, Lina Gega, Judith Gellatly, Adekeye Kolade, Diane Hunter, Craig Callender, Lesley-Anne Carter, Rachel Meacock, Peter Bower, Nicky Stanley, Rachel Calam, Miranda Wolpert, Paul Stewart, Richard Emsley, Kim Holt, Holly Linklater, Simon Douglas, Bryony Stokes-Crossley and Jonathan Green | 2020 | UK | No | YP took part only as research participants. |
| **87** | Co-designing a virtual world with young people to deliver social cognition therapy in early psychosis | Alba Realpe, Farah Elahi, Sandra Bucci, Max Birchwood, Ivo Vlaev, David Taylor, Andrew Thompson | 2018 | UK | No | It is not clear the number of YP involved throughout the process or their characteristics (e.g., age range and mean). |
| **88** | “If you don't speak from the heart, the young mob aren't going to listen at all”: An invitation for youth mental health services to engage in new ways of working | Michael Wright, Tiana Culbong, Nikayla Crisp, Britta Biedermann, Ashleigh Lin | 2019 | Australia | No | They just generically refer to “Aboriginal and Torres Strait Islander young people.” |
| **89** | Drafting the Aboriginal and Islander Mental Health Initiative for Youth (AIMhi-Y) App: Results of a formative mixed methods study | Josie Povey, Michelle Sweet, Tricia Nagel, Patj Patj Janama Robert Mills, Catherine Pumuralimawu Stassi, Anne Marie Ampirlipiyanuwu Puruntatameri, Anne Lowell, Fiona Shand, Kylie Dingwall | 2020 | Australia | No | YP took part only as research participants. |
| **90** | Service users as the key to service change? The development of an innovative intervention for excluded young people | Sally Zlotowitz, Chris Barker, Olive Moloney, Charlotte Howard | 2016 | UK | No | Even though they stated that the process was “led by young people”, YP were involved as research participants. |
| **92** | Priority setting in research: user led mental health research | Marjorie Ghisoni, Christine Ann Wilson, Karen Morgan, Bethan Edwards, Natalie Simon, Emma Langley, Helen Rees, Amanda Wells, Philip John Tyson, Phil Thomas, Allen Meudell, Frank Kitt, Brian Mitchell, Alan Bowen, Jason Celia | 2017 | UK | No | They just refer to “20 people with a lived experience of mental ill health” - no specification about their age is provided |
| **94** | Reflections, impact and recommendations of a co-produced ecological momentary assessment (EMA) study with young people who have experience of suicidality and psychiatric inpatient care | L. Dewa, S. Pappa, L. Mitchell, M. Hadley, J. Cooke, P. Aylin | 2022 | UK | No | Conference paper |
| **95** | Development of a co-produced tool for monitoring and supporting the mental health of young people | Joy MacKeith, Anna Good and Sara Burns Triangle | 2021 | UK | No | Conference paper |
| **96** | YOUNG SERVICE USERS, PROVIDERS AND RESEARCHERS COLLABORATE TO CO-PRODUCE A PREPARATION PROGRAMME FOR YOUNG PEOPLE LEAVING CHILD AND ADOLESCENT MENTAL HEALTH SERVICES AT 17/18 | V Dunn. | 2017 | UK | No | Conference paper |
| **97** | Developing a logic model for youth mental health: participatory research with a refugee community in Beirut | Rema A. Afifi, Jihad Makhoul, Taghreed El Hajj, Rima T. Nakkash | 2011 | Lebanon | No | YP took part only as research participants. |
| **98** | “I didn’t even know headspace had the drug thing until today”: A socio-ecological analysis of access to drug and alcohol interventions in integrated youth health care services | Nicole Snowdon, Julaine Allan, Anthony Shakeshaft, Ryan J. Courtney | 2023 | Australia | No | YP took part only as research participants. |
| **99** | Integrating human‐centred design into the development of an intervention to improve the mental wellbeing of young women in the perinatal period: the Catalyst project | Tatiana Taylor Salisbury, Katie H. Atmore, Inocencia Nhambongo, Muanacha Mintade, Luciana Massinga, Jak Spencer, Jonathan West, Flavio Mandlate | 2021 | Mozambique | No | YP took part only as research participants. |
| **100** | Adolescent perspectives on peripartum mental health prevention and promotion from Kenya: Findings from a design thinking approach | Tatiana Taylor Salisbury, Katie H. Atmore, Inocencia Nhambongo, Muanacha Mintade, Luciana Massinga, Jak Spencer, Jonathan West, Flavio Mandlate | 2024 | Kenya | No | YP took part only as research participants. |
| **101** | Strengthening mental health research outcomes through genuine partnerships with young people with lived or living experience: A pilot evaluation study | Grace Yeeun Lee, Sarah McKenna, Yun Ju C. Song, Alexis Hutcheon, Samuel J. Hockey, Rachael Laidler, Jo‐An Occhipinti, Claudia Perry, Tara Lindsay‐Smith, Annabel Ramsay, Skye Choi, Dakota Feirer, Andrew W. Shim, Jessica Cottle, Anith Mukherjee, Joshua New, Rebecca Yu, Elizabeth Mary Scott, Louise Freebairn, Ian Bernard Hickie | 2023 | Australia | No | They just mention “youth lived experience researchers”. No further detail about the age or demographics is provided. |
| **102** | The Need for Sensory-Friendly “Zones”: Learning From Youth on the Autism Spectrum, Their Families, and Autistic Mentors Using a Participatory Approach | Marc-André Clément, Keven Lee, Melissa Park, Anabel Sinn, Natalie Miyake | 2022 | USA | No | They just mention ‘youth’- no characterisation regarding age or other aspects. |
| **103** | Recommendations for improving adolescent addiction recovery support in six northwest tribal communities | Marc-André Clément, Keven Lee, Melissa Park, Anabel Sinn, Natalie Miyake | 2021 | USA | No | No information about YP - they just referred to “Tribal Council representatives”. |
| **104** | The BRAVE Study: Formative Research to Design a Multimedia Intervention for American Indian and Alaska Native Young Adults | Stephanie Craig Rushing, Allyson Kelley, Steven Hafner, David Stephens, Michelle Singer, Dyani Bingham, Colbie Caughlan, Bethany Fatupaito, Amanda Gaston, Thomas Ghost Dog, Paige Smith, Danica Love Brown, Celena McCray | 2021 | USA | No | YP took part only as research participants. |
| **106** | Evaluation of a longitudinal digital citizen science initiative to understand the impact of culture on Indigenous youth mental health: Findings from a quasi-experimental qualitative study | Susannah Walker, Prasanna Kannan, Jasmin Bhawra, Tarun Reddy Katapally | 2023 | Canada | No | YP took part only as research participants. |
| **107** | Promoting Engagement With Smartphone Apps for Suicidal Ideation in Y oung People: Development of an Adjunctive Strategy Using a Lived Experience Participatory Design Approach | Daniel Z Q Gan; Lauren McGillivray; Mark Erik Larsen; Taylor Bloomfield; Michelle Torok | 2023 | Australia | No | YP took part only as research participants. |
| **105** | Exploring young people’s experiences of the Tree Of Life narrative intervention through participatory research | Hannah Fleming, Amelia Paton & Janet Rowley | 2023 | UK | No | Does not focus on mental health |
| **108** | My Best Alaskan Life: Addressing Adolescent Mental and Reproductive Health in Alaska | Lauren Lessard, Cornelia Jessen, MA2 Sara L. Buckingham, PhD1 Riley Russell, MA, MS1 S. Abigail Morgan, MSN, WHNP-BC3 Jennifer Baker, BA4 | 2024 | USA | No | They mention ‘co-researchers’ and ‘participatory’ approaches, but no further details about age range and other YP characteristics are provided. The details available are regarding research participants. |
| **110** | Supporting mental health and psychosocial wellbeing through social and emotional learning: A participatory study of conflict- affected youth resettled to the U.S. | Lindsay Stark, Mackenzie V. Robinson, Alli Gillespie, Jeremy Aldrich, Wafa Hassan, Michael Wessells, Carine Allaf, Cyril Bennouna | 2021 | USA | No | Even though they refer to participatory methods, YP are involved just as research participants. |
| **111** | The Wellness Quest: A health literacy and self-­ advocacy tool developed by youth for youth mental health | Asavari Syan, Janice Y. Y. Lam, Christal G. L. Huang, Maverick S. M. Smith, Karleigh Darnay, Lisa D. Hawke, Joanna Henderson | 2020 | Canada | No | The range is not clear. They just refer to “age range is within the definition of youth suggested by Statistics Canada, that is, 15-29.” |
| **114** | A Social Media Group Cognitive Behavioral Therapy Intervention to Prevent Depression in Perinatal Y outh: Stakeholder Interviews and Intervention Design | Anupa Gewali, Alana Lopez, Kristin Dachelet, Elise Healy, Marimirca Jean-Baptiste, Holly Harridan, Yolanda Evans, Jennifer A. Unger, Amritha Bhat, Darius Tandon, Keshet Ronen | 2021 | USA | No | YP took part only as research participants. |
| **117** | Being visible: PhotoVoice as assessment for children in a school-based psychiatric setting | Vanessa Greco, Heather Christine Lambert & Melissa Park | 2016 | Canada | No | Children from 8 to 12 years old are involved as research participants. |
| **118** | Exploration of Psychological Well-Being, Resilience, Ethnic Identity, and Meaningful Events Among a Group of Youth in Northern England: An Autobiographical Narrative Intervention Pilot Study | Caroline S. Clauss-Ehlers | 2020 | UK | No | YP involved just as ‘research participants’ - despite referring to ‘participatory methods’ |
| **119** | ‘Something needs to change’: Mental health experiences of young autistic adults in England | Laura Crane, Fern Adams, Georgia Harper, Jack Welch, Elizabeth Pellicano | 2019 | UK | No | YP took part only as research participants. |
| **120** | Participatory Research Principles in Human-Centered Design: Engaging Teens in the Co-Design of a Social Robot | Elin A. Björling, Emma Rose | 2019 | UK | No | No age range provided. They just refer to ‘teens’. |
| **121** | The Optimization of an eHealth Solution (Thought Spot) with Transition-Aged Y outh in Postsecondary Settings: Participatory Design Research | Nicole VanHeerwaarden, Genevieve Ferguson, Alexxa Abi-Jaoude, Andrew Johnson, Elisa Hollenberg, Gloria Chaim, Kristin Cleverley, Gunther Eysenbach, Joanna Henderson, Andrea Levinson, Janine Robb, Sarah Sharpe, Aristotle Voineskos, David Wiljer | 2018 | Canada | No | YP took part only as research participants. |
| **122** | It's making us look disgusting…and it makes me feel like a mink…it makes me feel depressed!: using photovoice to help ‘see’ and understand the perspectives of disadvantaged young people about the neighbourhood determinants of their mental well-being | Megan Watson & Flora Douglas | 2012 | UK | No | YP took part only as research participants. |
| **124** | Development of a Peer Support Mobile App and Web-Based Lesson for Adolescent Mental Health (Mind Y our Mate): User-Centered Design Approach | Louise Birrell, Ainsley Furneaux-Bate, Jennifer Debenham, Sophia Spallek, Nicola Newton, Catherine Chapman | 2022 | Australia | No | YP took part only as research participants. |
