# Supplementary material for: Conceptualising and operationalising ‘patient and public involvement’ (PPI) and ‘responsible research and innovation’ (RRI) in youth mental health research: a scoping review
Source: Res Involv Engagem. 2026 Sep 24;12:156. doi: 10.1186/s40900-026-00969-3 (PMC13602541; doi:10.1186/s40900-026-00969-3)
Supplement: Supplementary file 3 [file 40900_2026_969_MOESM3_ESM.docx]

# **Additional File 3: Data Extraction Tool**

**Article:** Involving Young People in Mental Health Research: A Scoping Review of ‘Patient and Public Involvement’ (PP)I and ‘Responsible Research and Innovation’ (RRI) Approaches

**Journal:** Research Involvement and Engagement

**Authors:** Josimar Antônio de Alcântara Mendes, Mathijs Lucassen, Alex Adams, Ayan Mahamud, Lucy Hitcham, Sachiyo Ito-Jaeger, Christine Aicardi, Emma Nielsen, Lily Roberts, Ellen Townsend and Marina Jirotka

| **Category** | **Data Extracted** |
| --- | --- |
| *1. General descriptions and methods* | 1.1. Authors:_________  1.2. Year:_________  1.3. Place:_________  1.4. Collaborative Approach:  ☐ **PPI**  (The active collaboration of patients and the public in research, conducted with or by them, enriching the research process through their unique perspectives. This inclusive approach improves research quality and impact, with diverse roles and methods—such as communication, consultation, and participation—spanning from topic selection to practical implementation)  ☐ **RRI**  (It operates as an anticipatory, reflexive, and deliberative framework, where ethical and social implications are assessed from the initial design stages through to application, prioritising public interest and minimising harm. It also encourages inclusiveness and shared accountability, assessing ethical implications from design to application and minimising harm. Responsibility is distributed across stakeholders to support sustainable solutions to societal challenges)  ☐ Other: ____________  ☐ Unclear  1.5. How many young people involved? _____  1.6. Range age of young people involved:_________  1.7. Mean age of young people involved:_________  Type of design:  ☐ 1.8. Qualitative (descriptive, e.g., ethnography)  ☐ 1.9. Qualitative (documental)  ☐ 1.10. Qualitative (arts-based)  ☐ 1.11. Qualitative interviews with professionals (e.g., researchers and practitioners)  ☐ 1.12. Qualitative interviews with young people  ☐ 1.13. Qualitative interviews with young people and professionals  ☐ 1.14. Quantitative:_______  ☐ 1.15. Mixed Methods:_______  ☐ 1.16. Other:_______ |
|  |  |
| *2. Involvement characterisation* | 2.1. Study’s specific mental health topic: 2.2. Type and extensiveness of young people’s involvement: ☐ **Co-researchers/Co-developers** (When young people join project teams as 'lived experience' experts, collaborating alongside academics/professionals in all project phases – conception, planning, execution, and delivery. They are fully integrated into decision-making, ensuring their insights shape project outcomes directly) ☐ **Co-design** (It is a collaborative process that engages users, stakeholders, and designers as partners, integrating user insights to create relevant solutions. It values users as “experts of their experiences” by employing participatory methods like workshops and interactive activities to ensure design outcomes are informed by real-life perspectives and are both ethical and practical) ☐ **Consultation** (It gathers young people’s views to inform projects, services, or research by seeking their feedback on materials and policies. Unlike full participation, consultation usually involves input without direct decision-making or further participation. This approach values their perspectives, making resources more relevant and sometimes testing their suggestions in practical applications) ☐ Other:_________ 2.3. The process of collaboration was held: ☐ Online/Virtually ☐ In person ☐ Hybrid (both online/virtually and in person) ☐ Unclear 2.4. Are the young people co-author the paper? ☐ Yes (listed individually) ☐ Yes (listed as a YPAG/CYPAG) ☐ No ☐ Unclear |
| *3. Operational definitions for involvement* | 3.1. Study’s PPI definition, if any: 3.2. Study’s RRI definition, if any: |
|  | 3.3. Main advantages of collaborating with you people, if any: 3.4. Main challenges of collaborating with you people, if any: 3.5. Collaboration good practices, if any: |
|  |  |
