# Supplementary material for: Conceptualising and operationalising ‘patient and public involvement’ (PPI) and ‘responsible research and innovation’ (RRI) in youth mental health research: a scoping review
Source: Res Involv Engagem. 2026 Sep 24;12:156. doi: 10.1186/s40900-026-00969-3 (PMC13602541; doi:10.1186/s40900-026-00969-3)
Supplement: Supplementary file 4 [file 40900_2026_969_MOESM4_ESM.docx]

# **Additional File 4: Definitions (Excerpts)**

**Article:** Involving Young People in Mental Health Research: A Scoping Review of ‘Patient and Public Involvement’ (PP)I and ‘Responsible Research and Innovation’ (RRI) Approaches

**Journal:** Research Involvement and Engagement

**Authors:** Josimar Antônio de Alcântara Mendes, Mathijs Lucassen, Alex Adams, Ayan Mahamud, Lucy Hitcham, Sachiyo Ito-Jaeger, Christine Aicardi, Emma Nielsen, Lily Roberts, Ellen Townsend and Marina Jirotka

**DEFINITIONS FOR INVOLVEMENT APPROACHES IN ADOLESCENT MENTAL HEALTH**

**PPI:**

**P7:** “Patient and public involvement (PPI) is defined as research with and by patients, rather than to, for or about them. In recent years, there has been a significant shift in PPI in research, moving away from tokenism, towards more meaningful involvement and co-production. This has been facilitated by changes in research infrastructure and the growing patient voice. Increasingly, funding bodies have made it compulsory for prospective applicants to declare how patients, carers or the public have and will be involved in the proposed research. Likewise, some journals also require researchers to add a PPI statement3,4 to their manuscript. This has led to research organizations creating dedicated PPI resources

within their institutions”. (…) PPI is ultimately about an interaction between researchers and patients or members of the public, which is dynamic, context specific and dependent upon many uncontrollable factors” (p. 135)

**P26:** “Patient and public involvement (PPI) is defined as research being carried out ‘with’ or ‘by’ patients and members of the public rather than ‘to’, ‘about’ or ‘for’ them. (…) PPI activities, such as co-design and co-production of resources, have been demonstrated to greatly improve the acceptability of interventions and processes in research” (pp. 2-3)

**Co-creation:**

**P1:** “Co-creation process shifts away from the traditional method of involving passive stakeholders during the latter phase of prototype testing towards viewing them as active contributors with knowledge and skills for co-creation during the ideation phase” (pp. 3-4)

**Co-production:**

**P7:** “Principles of Co-production: 1. Sharing of power; 2. Including all perspectives and skills; 3. Respecting and valuing knowledge of all those working together on the research; 4. Reciprocity; and 5. Building and maintaining relationships” (p. 138)

**P12:** “Co‐production as outlined by the NIHR involves five core principles: sharing of power; building and maintaining relationships; including all perspectives and skills; respecting and valuing each others' knowledge and reciprocity” (p. 3144)

**P15:** “Coproduction was originally developed as an approach to service development where adult service users were recognized as active participants in shaping and delivering services rather than as passive recipients of services. (…) Within mental health research specifically, coproduction is a relatively new and innovative approach, with evidence of positive involvement and outcomes including improved mental well-being for people involved in the process” (p. 2)

**P24:** “[co-production] is defined as a way of doing research in which ‘researchers, practitioners and members of the public work together, sharing power and responsibility from the start to the end of the project’ (…) It is expected to drive more egalitarian, democratic or transparent research processes, which more effectively address the needs of patients, service users and/or marginalised citizens. In a co-production process, different stakeholders bring their skills, life experiences and social roles to the table. Together they develop the various stages of research, such as idea generation, funding acquisition, study design, management, data collection, analysis, evaluation and dissemination” (p. 2)

**P31:** “Coproduction, also referred to as transformation of power relations in research/practice, is a relatively new concept, something that is more commonly associated with health and social care practice, rather than research. A coproductive approach to research involves collaborations between service users, practitioners and academics/researchers, as allies, to produce transformative and meaningful outcomes. As such, coproduced research allows for the redressing of power imbalances, providing a foundation for relational ethics and confronting complexities head-on, as will be seen later on in this article. In line with the key principles of coproduction, namely inclusivity/diversity, respecting knowledge and reciprocity, we involved young people and practitioners in all the stages of our project, from early conceptualisations of key terms, through to choices about methodology. (…) can promote justice, giving a voice to young people as valued participants in the coproduction of knowledge, thereby fundamentally democratizing the relationships between different parties: researchers, young people and

Practitioners. The result may be messy, yet engaging with these challenges has enormous potential for making sense of (and centralising) young people’s lived experiences of mental health and wellbeing” (p. 93)

**Co-design:**

**P18:** “Co-design aims to establish end-users’ needs and preferences and ensures that intervention content, features and aesthetics align to these. For young people, this involves ensuring the intervention is appealing, age-appropriate, relatable and suitable to their lifestyle” (p. 3)

**P22:** “co-design refers to “the process in which actors share their knowledge about both the design process and the design content…to create shared understanding on both aspects…and achieve the larger common objective: the new product to be designed” (p. 2)

**P23:** “Codesign is a best practice for DMHI [Digital Mental Health Interventions] design that underpins successful adoption and implementation and extends reach to marginalized populations. Codesign of DMHIs centers lived experience through iterative engagement with end users, referring to the intended recipients of an intervention, and additionally considers the lived experiences of those within end users' support systems. (…) For codesign of DMHIs, end-users' ideologies toward technology should also be considered, in recognition that one's culture and experiences can influence comfort with using technology for health-related purposes” (p. 2)

**P27:** “Co-design is a crucial component of research and development, referring to the meaningful engagement of relevant stakeholders in the design process. It involves seeking out the lived experiences of potential end-users and their networks, understanding their needs and preferences, and taking into consideration contextual factors, in order to design tools, interventions or products that facilitate end-user engagement and uptake. (…) Co-design goes beyond consultation and observation, requiring researchers and designers to actively collaborate

with end-users to design solutions.38 Co-design may incorporate a range of activities to engage young people, such as discussions, storytelling, mapping exercises, drawing, and prototype feedback. Co-design approaches with young children and adolescents should ensure that research activities are engaging, age appropriate (e.g., consideration of reading abilities), and culturally relevant” (p. 2-3)

**Participatory Research:**

**P2:** “Treating research as a shared exercise between researcher and patient shifts the balance of power so that it becomes more equal. In a participatory research project this is done through reflexivity, disclosure and involving patients as coresearchers. This model of participatory

research is often referred to as ‘cooperative enquiry’ between coresearchers with different perspectives resulting in new interpretations” (p. 858)

**P4:** “Participatory research (PR) approaches, rooted in community development, are democratic, collaborative and aimed to redress the researcher/subject power imbalance. Under the PR umbrella sits a range of innovative, creative and arts-based techniques which do not rely exclusively on verbal or written competencies. These less conventional approaches recognise that‘some knowings cannot be conveyed through language’ and may be particularly appropriate when working with more vulnerable groups to facilitate meaningful exploration of complex or sensitive material” (p. 2)

**Single-session Intervention (SSI):**

**P21:** “[it] may help address the need for accessible, easily completable, and effective mental health interventions for adolescents. [SSI is] defined as “structured programs that intentionally involve only one visit or encounter with a clinic, provider, or program; they may serve as stand-alone or adjunctive clinical services”. [SSI] can take multiple forms (eg, provider delivered and self-guided); however, the flexible and scalable nature of digital, self-administered SSIs allows them to be delivered and completed in low-resource settings relatively rapidly and broadly. Existing literature supports the effectiveness of SSIs for adolescents’ depression and anxiety, even when delivered as stand-alone mental health supports” (p. 2)

**Co-operative Enquiry:**

**P3:** “This entailed working ‘with’ rather than ‘on’ young people. (…) Co-operative enquiry often explores ways of transforming processes and procedures (on personal and organisational levels) and identifying best practice though cycles of reflection and action” (p. 173)

**Design Thinking:**

**P8:** “[It] was modeled on a Double-Diamond Design Thinking process that comprises four phases of exploration and execution: issues were discovered, clearly defined and prioritised, potential interventions developed and selected, and prototyped interventions delivered” (p. 30)

**P14:** “Design Thinking can be seen as a participatory approach that is an iterative process, which involves the end-users in the co-creation and evaluation of digital resources, including eMental health screening app” (p. 4)

**Arts-based Methods:**

**P22:** “Participatory in nature, arts-based methods (ABM) have been defined as “research methods in which art forms play a primary role in any or all of the steps of the research process” (p. 1)

**Participatory Design:**

**P5:** “Participatory design (PD) methodologies, which were developed in the late 1960s and early 1970s, emphasize the importance of

involving all stakeholders (including end users, developers, and researchers) during the development of products to help ensure the end product meets everyone’s needs; improve usability; and increase engagement of users. The process involves iterative design cycles in which end users and researchers contribute to knowledge production and the development of the end product. Importantly, end users should participate in all stages of development, not as consultants or controllers of the process, but sharing equal responsibility with the research team for the outcomes. Some researchers, including us, consider PD as key research methodologies that overtly put the end user at the center of research and here, the development of the MHeC” (p. 3)

**P9:** “[Participatory Design (PD) is a solution that] can derive maximum value from (…) interventions, it is critically important to involve them in their design and development and to strike a balance between best clinical practice and user experience (including usability). (…) The process [of PD] involves engaging end users and other stakeholders at all stages (from conception to completion) of the design, development, and testing of these technologies. Through several iterative phases, the prototype is co-designed, codeveloped, and refined until it has value to the end users; meets their needs; and is appealing, engaging, acceptable, and usable. As end users share equal responsibility with the researchers for the outcomes, the rationale behind the use of PD methodologies could result in better products that are more functional in real-life settings, thereby closing the translational research gap” (p. 3)

**Agile Development (System/Software):**

**P19:** “agile system development – a set of practices in which requirements are gathered and the software is developed, tested, and improved in an iterative process”. (…) agile software development is a methodology in which requirements are gathered and the software is developed, tested, and further improved in an iterative process. The team that develops the software includes developers and potential users, an approach that is also known as participatory design (p. 4)

**Discover, Design, Build, and Test (DDBT):**

**P23:** [DDBT] “provides a structure for DMHI development that features codesign with end-users. DDBT brings together principles of human centered design n (HCD) through incorporating iterative design and development, participatory action research by centering partnerships with end-users to bring about meaningful change with them rather than for them, and implementation science through a focus on understanding how best to implement and sustain a DMHI” (p. 2 )

**Community-based Participatory Research (CBPR):**

**P32:** “participants are engaged throughout the research process, such as collecting data, coanalyzing the data with researchers, and making key decisions in sharing the research findings” (p. 370)
