# Supplementary material for: Conceptualising and operationalising ‘patient and public involvement’ (PPI) and ‘responsible research and innovation’ (RRI) in youth mental health research: a scoping review
Source: Res Involv Engagem. 2026 Sep 24;12:156. doi: 10.1186/s40900-026-00969-3 (PMC13602541; doi:10.1186/s40900-026-00969-3)
Supplement: Supplementary file 5 [file 40900_2026_969_MOESM5_ESM.docx]

# **Additional File 5: ChatGPT Prompt for Definition Synthesis**

**Article:** Involving Young People in Mental Health Research: A Scoping Review of ‘Patient and Public Involvement’ (PP)I and ‘Responsible Research and Innovation’ (RRI) Approaches

**Journal:** Research Involvement and Engagement

**Authors:** Josimar Antônio de Alcântara Mendes, Mathijs Lucassen, Alex Adams, Ayan Mahamud, Lucy Hitcham, Sachiyo Ito-Jaeger, Christine Aicardi, Emma Nielsen, Lily Roberts, Ellen Townsend and Marina Jirotka

The process to synthesise the RRI and PPI concepts via ChatGPT involved two parts. The first part, ‘***Training and Auditing***’, involved instructing and refining the task. The second part, ‘***Running the Definitions***’, involved sharing the original excerpts and asking ChatGPT to synthesise the concepts accordingly.

**PART 1 (TRAINING AND AUDITING):**

**1º Prompt to ChatGPT:**

I will provide 4 different definitions for ‘Patient and Public Involvement’ (PPI) alongside their sources. Your task is: 1) take into account the 4 definitions; 2) provide a summary definition for PPI based on those 4 definitions - this summary must consider and address the information from all 4 definitions; 3) for each sentence, you should indicate the sources you considered to write it. This will be needed to achieve point 4 - for instance: “given sentence [superscript indicating the source used]”; 4) provide a table with two columns. In the first, there will be the summary definition (as instructed in point 3). The second column there will be the sources.

- ***Definition 1:*** “a broad definition of public involvement of “research being carried out ‘with’ or ‘by’ members of the public rather than ‘to’, ‘about’ or ‘for’ them””. Source: Papageorgiou, V., Dewa, L. H., Bruton, J., & et al. (2023). 'Building bridges’: Reflections and recommendations for co-producing health research. Research Involvement and Engagement, 9, 113.

- ***Definition 2:*** “Patient and public involvement is thought to improve the way the research is prioritized, commissioned, undertaken, communicated and used. Active involvement of service users in research can lead to research of greater quality and relevance owing to the unique perspective that users can bring to a research project”. Source: Brett, J. O., Staniszewska, S., Mockford, C., Herron‐Marx, S., Hughes, J., Tysall, C., & Suleman, R. (2014). Mapping the impact of patient and public involvement on health and social care research: a systematic review. Health expectations, 17(5), 637-650.

- ***Definition 3:*** “controversy around the different labels used is unsurprising, as they are derived from a multitude of contexts and interests. They suggest that the term ‘lay’ is to be preferred, as it denotes a broader alternative perspective to that of the professional. What is clear is that no single label will be acceptable to everyone, and we took a pragmatic approach by agreeing with the RAPPORT PPI representatives what terms should be used within the report. We use two terms interchangeably: ‘PPI representatives’ to emphasise individuals undertaking diverse roles from patient representative through to a member of the general public, and ‘lay representatives’ to emphasise the overall perspective these individuals bring”. Source: Wilson, P., Mathie, E., Keenan, J., McNeilly, E., Goodman, C., Howe, A., ... & Peckham, S. (2015). ReseArch with Patient and Public invOlvement: a realisT evaluation: the RAPPORT study. Health services and delivery research.

- ***Definition 4:*** “PPIP refers to at least one formal method of involving patients and the public in CPG development and/or implementation. Involvement methods may include: communication (information is communicated to patients/the public); consultation (information is collected from patients/the public); or participation (patients/the public exchange information with other stakeholders). PPIP can be used at different stages, from the macro-level of CPG development (eg, topic selection, evidence review, recommendation, and development of ancillary products), its meso-level of implementation to specific target groups, or its use at the micro-level of the clinical consultation”. Source: Boivin, A., Currie, K., Fervers, B., Gracia, J., James, M., Marshall, C., ... & Burgers, J. (2010). Patient and public involvement in clinical guidelines: international experiences and future perspectives. Quality and Safety in Health Care, 19(5), 1-4.

***ChatGPT’s output:***

**2º Prompt to ChatGPT:**

[This is the output deriving from the 1º prompt about]

***ChatGPT’s output:***

The prompt you’re referring to was developed as a **summary definition paragraph** for *Patient and Public Involvement (PPI)*, based on four provided academic definitions. Here's an analysis and recap of the **parameters** and **principles** I followed to construct that response:

**✅ Purpose of the Prompt**

To produce a **concise, coherent, and evidence-based definition** of PPI that:

- Synthesises **four distinct scholarly definitions** you provided;
- Preserves the **unique contributions of each definition**;
- Uses **clear academic language**;
- Assigns **citation markers ([1], [2] etc.)** to indicate which sources informed each sentence;
- Presents the full definition as a **single paragraph**, as explicitly requested.

**🔍 Parameters Used in the Prompt**

**1. Source Integration Strategy**

Each sentence in the paragraph was constructed by synthesising content across one or more definitions you provided. Here's how that was managed:

- **Sentence 1**: Defined PPI as active, collaborative involvement — drawn primarily from Papageorgiou et al. [1] and Brett et al. [2].
- **Sentence 2**: Emphasised the value of lived experience and its impact on research quality — based on Brett et al. [2].
- **Sentence 3**: Explained the terminology around “PPI representatives” and “lay representatives” — from Wilson et al. [3].
- **Sentence 4**: Described the scope and forms of involvement across stages — based on Boivin et al. [4].

**2. Content Prioritisation**

Key elements included:

- **Nature of involvement**: research conducted *with or by*, not *to or for* the public.
- **Benefits**: improving quality, relevance, and applicability of research.
- **Terminology and role diversity**: acknowledging varied identities within “the public”.
- **Levels and methods**: communication, consultation, participation across research phases.

**3. Language and Tone**

- **Academic and accessible**: Technical but readable, aligning with expectations for peer-reviewed publications.
- **Objective and neutral**: Avoided normative claims or advocacy language.
- **Precise structure**: Each sentence built upon the previous to support logical progression.

**4. Formatting and Structure**

- Paragraph was delivered as a **single, coherent block** (as requested);
- **In-text citation markers** ([1], [2], etc.) matched the original source order you provided.

**Summary of Parameters**

| **Element** | **Parameter** |
| --- | --- |
| Paragraph Structure | Single paragraph, logical flow |
| Citation Style | Numeric markers [1], [2], [3], [4] |
| Source Attribution | One or more sources per sentence |
| Language | Formal, academic, accessible |
| Integration Focus | Synthesis of overlapping and complementary points across sources |
| Content Focus | Definition, scope, terminology, value, levels of engagement |
| Use of Original Content | No invention; all points grounded in definitions you had provided |
| Format Request Honoured | Paragraph, not a bulleted or line-separated format |

**PART 2 (RUNNING THE DEFINITIONS):**

**3º Prompt to ChatGPT:**

Great, considering these parameters, I will need your help to synthesise some definitions. For this, I will share: a) the concept title; b) the scholarly definition excerpts; and c) the source. Your task is to follow the format that you just recapped and present the full definition as a single paragraph with citation markers ([P1], [P2] etc.). Use the same language, tone, formatting and structure you just recapped. Important: do not use Python or Canvas or consider any other source of information that is not the scholarly definition excerpts that I provide!

> Concept title: [given concepts]

> Scholarly definition excerpts: [related excerpts – shown in Addition File 4]
